# Alterations in Glycerolipid and Fatty Acid Metabolic Pathways in Alzheimer's Disease Identified by Urinary Metabolic Profiling: A Pilot Study

**DOI:** 10.3389/fneur.2021.719159

**Published:** 2021-10-27

**Authors:** Yumi Watanabe, Kensaku Kasuga, Takayoshi Tokutake, Kaori Kitamura, Takeshi Ikeuchi, Kazutoshi Nakamura

**Affiliations:** ^1^Division of Preventive Medicine, Graduate School of Medical and Dental Sciences, Niigata University, Niigata, Japan; ^2^Department of Molecular Genetics, Brain Research Institute, Niigata University, Niigata, Japan; ^3^Department of Neurology, Brain Research Institute, Niigata University, Niigata, Japan

**Keywords:** targeted metabolomics, targeted lipidomics, CE-TOFMS, LC-FTMS, urine, biomarker, Alzheimer's disease

## Abstract

An easily accessible and non-invasive biomarker for the early detection of Alzheimer's disease (AD) is needed. Evidence suggests that metabolic dysfunction underlies the pathophysiology of AD. While urine is a non-invasively collectable biofluid and a good source for metabolomics analysis, it is not yet widely used for this purpose. This small-scale pilot study aimed to examine whether the metabolic profile of urine from AD patients reflects the metabolic dysfunction reported to underlie AD pathology, and to identify metabolites that could distinguish AD patients from cognitively healthy controls. Spot urine of 18 AD patients (AD group) and 18 age- and sex-matched, cognitively normal controls (control group) were analyzed by mass spectrometry (MS). Capillary electrophoresis time-of-flight MS and liquid chromatography–Fourier transform MS were used to cover a larger range of molecules with ionic as well as lipid characteristics. A total of 304 ionic molecules and 81 lipid compounds of 12 lipid classes were identified. Of these, 26 molecules showed significantly different relative concentrations between the AD and control groups (Wilcoxon's rank-sum test). Moreover, orthogonal partial least-squares discriminant analysis revealed significant discrimination between the two groups. Pathway searches using the KEGG database, and pathway enrichment and topology analysis using Metaboanalyst software, suggested alterations in molecules relevant to pathways of glycerolipid and glycerophospholipid metabolism, thermogenesis, and caffeine metabolism in AD patients. Further studies of urinary metabolites will contribute to the early detection of AD and understanding of its pathogenesis.

## Introduction

Alzheimer's disease (AD) is the most common form of dementia, accounting for 50–70% of cases. It is a slowly progressive neurodegenerative disease that is thought to begin 20 years before symptoms such as memory and language problems are noticed by individual patients (1). Since there is no cure to date, a preventative approach before clinical onset is emphasized (2). Biomarkers that can predict the onset of AD are highly desirable, and such biomarkers should ideally be non-invasive, easy to apply to asymptomatic or very early-stage patients, and reflect physiological changes associated with the disease.

The main neuropathological changes associated with AD are the extracellular accumulation of β-amyloid plaques, intracellular accumulation of neurofibrillary tangles of tau protein, inflammation, and brain atrophy (1). However, the disease etiology is not completely understood. Increasing evidence indicates that both peripheral and central metabolic dysfunction underly AD pathophysiology (3, 4).

Metabolomics is a discipline that comprehensively analyzes all small molecules and metabolites in cells, tissues, and biofluids under a given set of conditions (5). A concentration change in certain sets of metabolites may provide a global overview of multiple relevant biochemical pathways, reflecting changes in downstream biological processes (6). Mass spectrometry (MS) and NMR spectrometry are two primary platforms for metabolomics analysis which can be used to measure various molecules in biological materials, with MS being the more commonly used modality (7, 8). In MS, the coverage of detectable metabolites varies greatly depending on the separation analyzer used to separate the sample and the type of mass spectrometer used for subsequent detection (9).

Urine is a highly desirable source of disease biomarkers, as it is collectable in large volumes non-invasively. Urine contains components from plasma glomerular filtration and excretion from the renal tubule and urogenital tract. Thus, it reflects the metabolic and pathophysiological conditions of an individual (10). Due to the glomerular filtration barrier, urine is largely free from proteins and lipids that can interfere with measurements. Consequently, compounds that are far below the limit of detection in blood are well above the detection limit in urine (11), providing a richer material for analysis. Metabolomics has been used to explore urinary metabolic profiles of various diseases, such as chronic kidney disease (12), urological and non-urological cancers (13), polycystic ovary syndrome (14), ischemic stroke (15), gestational diabetes mellitus (16), asthma (17), mood disorders and schizophrenia (18), and Parkinson's disease (19, 20).

Revealing the urinary metabolic profile of AD is of particular interest because it may provide global pathophysiologic information on the disease and yield non-invasive biomarker candidates. Several studies have reported on the urinary metabolite profile of AD patients (21–24). For instance, Cui et al. identified 12 metabolites significantly increased in AD patients mainly reflecting metabolic changes in fatty acids and amino acids, and identified urine 5-L-glutamylglycine as a candidate biomarker (21). Yilmaz et al. identified 11 metabolites that were significantly altered in AD patients (24). Kurbatova et al. identified a set of 32 metabolites strongly associated with AD which suggested the involvement of pathways of cholesterol metabolism, gut microbiota, DNA methylation, polyamine metabolism, and insulin resistance (23). Whiley et al. reported significantly lower metabolite concentrations of tryptophan pathway metabolites in AD patients relative to cognitively normal controls (22). Nevertheless, given the diversity of metabolites in urine, more research using various measurement platforms is needed.

Since metabolites exhibit a variety of chemical and physical properties, it is currently not possible to analyze the entire metabolome on a single analysis platform. In this study, we used capillary electrophoresis time-of-flight mass spectrometry (CE-TOFMS) and liquid chromatography-Fourier transform mass spectrometry (LC-FTMS) to cover a larger range of molecules with hydrophilic (ionic) (25, 26) and hydrophobic (lipid) characteristics (27, 28). This allowed us to explore and compare a more comprehensive metabolome of urine samples from AD patients and cognitively normal, age- and sex-matched controls. The purpose of this pilot study was to examine whether the metabolic profile of urine from AD patients reflects the metabolic dysfunction reported to underlie AD pathology, and to explore possible metabolites that could discriminate AD patients from cognitively healthy controls.

## Materials and Methods

### Participants

This study was approved by the human research ethics committee of Niigata University (approval number: 1836, 2015-2081). All participants from Niigata University Hospital signed informed consent forms, and all participants of the Murakami cohort (29) were informed through a verbal consent process.

Participants were 18 AD patients and 18 cognitively healthy individuals selected in an age- and sex-matched manner. Participant recruitment was described previously (30). Briefly, AD patients were recruited from among outpatients of Niigata University Hospital who were diagnosed with the disease based on criteria of the National Institute on Aging-Alzheimer's Association (NIA-AA) and took the Mini-Mental State Examination (MMSE) (31) within a year of urine collection. Cognitively normal, age- and sex-matched controls (MMSE score >27) were selected from a subcohort (Sekikawa cohort) of the Murakami cohort, a population-based cohort study targeting areas of northern Niigata Prefecture (Murakami region) (29). Participants provided spot urine samples at specific health checkups held by the national health insurance of Japan (control group) or during outpatient visits (AD group), and took the MMSE within a year of urine collection. No restrictions on diet, drinking, or exercise were required prior to urine sampling. The procedures for urine collection and storage can be found in the online Supplementary Material.

Participant characteristics were described previously (30), and clinical characteristics of patients are summarized in Supplementary Table 1. Briefly, mean ages of AD and control groups were 72.9 ± 5.6 and 72.8 ± 5.2 years, respectively, and there were 8 males and 10 females in each group. Mean MMSE scores were 21.6 ± 4.5 and 28.8 ± 0.7, respectively (*p* < 0.001, *t*-test). Disease duration of the AD group was 4.8 ± 2.5 years. All but one participant in the AD group had been prescribed anti-AD drugs (cholinesterase inhibitors and/or N-methyl-D-asparate receptor antagonist). Mean BMIs of the AD group and control group were 21.2 ± 2.9 m/kg^2^ (*n* = 11) and 23.7 ± 2.5 m/kg^2^ (*n* = 18), respectively, (*p* = 0.022, *t*-test).

### Urinary Metabolomics and Lipidomics

Detailed methods of urine sample collection, metabolomics and lipidomics analysis, and data processing can be found in the online Supplementary Material. Briefly, analyses of ionic metabolites were performed at Human Metabolome Technologies Inc. (HMT, Tsuruoka, Japan) using CE-TOFMS. Lipidomics analysis was performed at Chemicals Evaluation and Research Institute, Japan (CERI, Saitama, Japan) using LC-FTMS (28, 32).

The peak area of each metabolite was normalized to the creatinine concentration of each sample. Relative peak areas (normalized to the creatinine concentration) from CE-TOFMS and LC-FTMS, and information regarding the certainty of identification of detected peaks from LC-FTMS, are available in the Supplementary Datasets.

### Lipid Nomenclature

Lipids were abbreviated as follows: acyl carnitine (AcCa), bis-methyl phosphatidic acid (BisMePA), phosphatidylcholine (PC), phosphatidylethanolamine (PE), phosphatidylserine (PS), triacylglycerol (TG), ceramides phosphate (CerP), ceramides (Cer), sphingomyelin (SM), diacylglycerol (DG), fatty acid (FA), and monohexosylceramide (Hex1Cer). Acyl-chain structures are denoted as carbon chain length:number of double bonds, and are provided for each chain. Alkenyl bonds (plasmalogen type linkage) and alkyl bonds identified in glycerophospholipids were denoted with the ‘p’ suffix and ‘e’ suffix, respectively.

### Statistical Analysis

Wilcoxon's rank-sum test was used to compare levels of compounds between the AD group and control group. SAS software was used for statistical analyses (release 9.13, SAS Institute Inc., Cary, NC, USA, RRID:SCR_008567). *P* < 0.05 was considered statistically significant. The false discovery rate was controlled using Storey's method.

To identify metabolites which can discriminate the AD group from the control group, orthogonal partial least-squares discriminant analysis (OPLS-DA) was applied using SIMCA software (version 14.0, Umetrics AB, Umea, Sweden, RRID:SCR_014688). OPLS-DA performs supervised clustering which classifies AD patients and controls into two groups. Before OPLS-DA, data that were not normally distributed were logarithmically transformed accordingly with the automatic transformation criteria of the software. Data were then mean-centered and scaled to unit variance for equal metabolite weighting. The reliability of the models was determined by analysis of variance testing of cross-validated predictive residuals (CV-ANOVA). The cross validation was performed seven times. Variable importance in the projection (VIP) provides the influence of every variable in the model. A higher VIP value represents a stronger contribution to discrimination among groups. Variables with VIP > 1 have an above average influence on the model.

### Pathway and Heatmap Analysis

Kyoto Encyclopedia of Genes and Genomes (KEGG) compound database entries (RRID:SCR_012773) were searched for metabolites and mapped on KEGG pathways using KEGG Mapper (KEGG PATHWAY Database, RRID:SCR_018145).

MetaboAnalyst was used for pathway enrichment analysis and pathway topology analysis. KEGG ID was used for compound name mapping. Concentration data of significantly differentiated metabolites were uploaded, log-transformed, and auto-scaled prior to analysis. The selected pathway enrichment analysis method was Global test. Selected node importance measure for the topological analysis was relative betweenness centrality. AcCa, Mannosamine, and Hex1Cer were not included in the analysis, as they were not registered in the software internal database. When metabolites share the same KEGG ID, metabolites with earlier list order, that is, metabolites with smaller *p*-values in the bivariate analysis, were adopted.

Data processed for OPLS-DA analysis were used for heatmap-graph presentation. Graphs were made using GraphPad Prism (version 9, GraphPad Software, LLC., CA, USA, RRID:SCR_002798).

Processed data for bivariate analysis, and auto-transformed and scaled data for OPLS-DA analysis are available as Supplementary Datasets.

## Results

Urinary metabolites including both ionic and lipid molecules were identified, and their relative concentrations were compared between the AD group and control group. A total of 304 ionic molecules and 81 lipid compounds of 12 lipid molecular classes were identified using CE-TOFMS and LC-FTMS, respectively. Compounds with >50% missing values were excluded, and missing values were replaced by 1/5 of the minimum positive value for each compound, resulting in 198 ionic molecules and 81 lipids for further analysis. Twenty-six of the 279 metabolites showed significantly different relative concentrations between the two groups (*p* < 0.05), with 21 having lower concentrations in the AD group than in the control group, and five having higher concentrations in the AD group than in the control group (Table 1).

**Table 1:** Correlation coefficients for SC thickness values calculated by five different methods.

**Compounds**	**KEGG compound ID**	**Median fold change (AD/control)**	***p*-value (Wilcoxon)**	***Q*-value**	**VIP (Model 1)**
**Compounds significantly lower in AD urine compared to control urine**					
Glycerol 3-phosphate	C00093	0.44	0.001	0.111	2.732
Caffeine	C07481	0.05	0.002	0.142	2.158
Ethanolamine phosphate	C00346	0.51	0.002	0.142	2.379
Paraxanthine	C13747	0.46	0.004	0.197	2.112
Pimelic acid	C02656	0.65	0.007	0.285	2.492
AcCa (11:1)	C02301	0.45	0.010	0.296	2.535
AcCa (9:0)	C02301	0.54	0.013	0.340	2.035
DG (18:2_21:0)	C00165, C00641	0.46	0.018	0.375	1.533
Taurine	C00245	0.68	0.018	0.375	1.770
TG (16:0_16:0_16:0)	C00422	0.67	0.022	0.375	1.639
Mannosamine	C03570	0.74	0.033	0.375	2.058
Indole-3-acetaldoxime, indole-3-acetamide	C02937, C02693	0.63	0.036	0.375	1.725
TG (10:0_20:1_20:5)	C00422	0.53	0.038	0.375	1.431
Leucine	C00123, C01570, C16439	0.81	0.041	0.375	1.903
Piperidine	C01746	0.40	0.045	0.375	1.320
FA (21:0)	C00162, C00638	0.50	0.045	0.375	1.596
FA (14:0)	C00162, C06424	0.68	0.045	0.375	1.487
FA (36:0)	C00162	0.51	0.048	0.375	1.581
FA (33:0)	C00162	0.52	0.048	0.375	1.620
FA (30:0)	C00162	0.53	0.048	0.375	1.581
4-Guanidinobutyric acid	C01035	0.72	0.050	0.375	1.096
**Compounds significantly higher in AD urine compared to control urine**					
Hex1Cer (m19:0_21:2)	C05005	1.99	0.009	0.296	1.839
PE (16:0p_20:4)	C04756	1.39	0.028	0.375	1.445
PC (18:0_20:3)	C00157	1.50	0.035	0.375	1.583
PE (18:0p_20:4)	C04756	1.50	0.035	0.375	1.904
cAMP	C00575	1.26	0.037	0.375	0.857

PC and PE classes included molecules with diacyl, alkyl, and alkenyl side chains. These molecules were subclassified as diacyl, alkyl, and alkenyl groups, and total amounts of molecules in each lipid class and subclass were compared (Supplementary Table 5), provided that the main ions of the lipid molecules identified in each lipid class were identical (Supplementary Table 6). Total amounts of AcCa, DG, and FA were significantly lower in AD urine, and that of alkenyl PE was significantly higher in AD urine.

**Figure 1:**
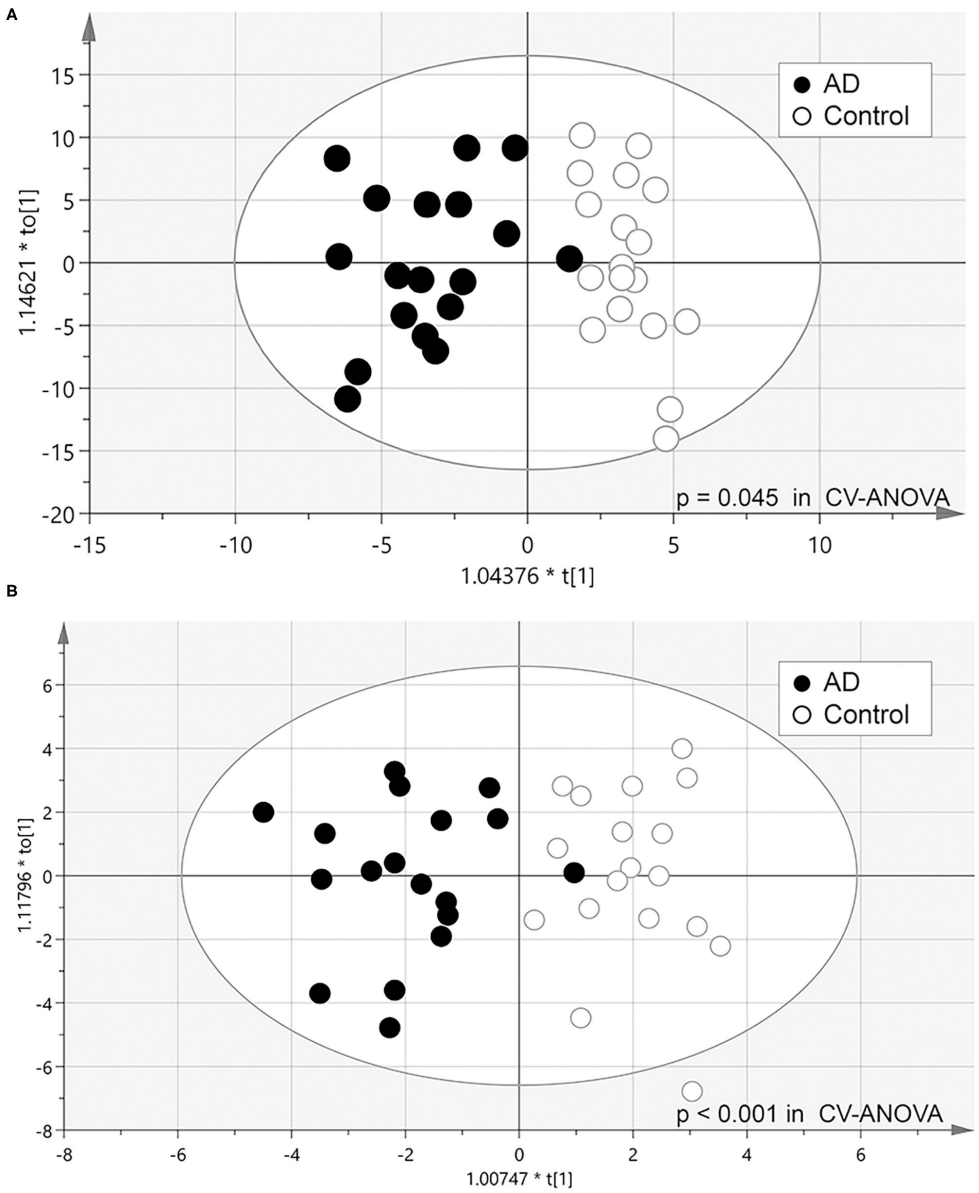
Scatter plot of OPLS-DA analysis. **(A)** Model 1: model constructed from all 279 metabolites identified (Model 1). **(B)** Model 2: model constructed from 26 metabolites that significantly differed in Wilcoxon's rank-sum test (*P* < 0.05).

To clarify the metabolic profile characteristics of AD urine, we attempted to discriminate between the AD group and control group using OPLS-DA. First, the relative concentration of all 279 metabolites were analyzed, resulting in a considerable separation between the AD group and control group (*p* = 0.045 in CV-ANOVA, Model 1) (Figure 1A). Next, the model which included 26 molecules significantly higher or lower in AD urine was able to more effectively discriminate the two groups in OPLS-DA (*p* < 0.001 in CV-ANOVA, Model 2) (Figure 1B). Further removal of metabolites did not increase model efficiency.

To assess the biological relevance of the 26 metabolites which significantly differed by relative amount between the two groups, these metabolites were annotated to KEGG compound IDs (Table 1) and mapped on KEGG pathways. KEGG IDs for some molecules were redundant due to the limitation in isomer separation and limited coverage of the database. The list of KEGG pathways that include four or more of the significantly differentiated metabolites in AD urine are shown in Table 2. The location of these metabolites in each pathway map and a heatmap representation of all metabolites included in each pathway are shown in Supplementary Figures 1 and 2, respectively.

**Table 2:** List of KEGG pathways that include 4 or more of the significantly differentiated metabolites in AD urine.

**Pathway ID**	**Description (Number of included metabolites)** **Included metabolites**
hsa01100	Metabolic pathways (15) Glycerol 3-phosphate, Leucine, PC, Taurine, Ethanolamine phosphate, TG, cAMP, DG, 4-Guanidinobutyric acid, Pimelic acid, Indole-3-acetaldoxime, Indole-3-acetamide, Ethanolamine plasmalogen, Caffeine, Paraxanthine
hsa04714	Thermogenesis (5) AcCa, TG, DG, FA, cAMP
hsa00561	Glycerolipid metabolism (4) Glycerol 3-phosphate, DG, TG, FA
hsa00564	Glycerophospholipid metabolism (4) Glycerol 3-phosphate, Ethanolamine phosphate, PC, DG
hsa05231	Choline metabolism in cancer (4) Glycerol 3-phosphate, PC, FA, DG
hsa04923	Regulation of lipolysis in adipocytes (4) FA, DG, TG, cAMP

Pathway enrichment and pathway topology analysis using the relative concentration values of metabolites was performed using Metaboanalyst to identify pathways that are highly relevant to the components of AD urine. AcCa, Mannosamine, and Hex1Cer were not included in the analysis because they were not in the software database. Pathways that were significantly enriched after adjustment by the Holm-Bonferroni method are shown in Table 3. Among these significantly enriched pathways, the caffeine pathway had the strongest impact (Table 3).

**Table 3:** Significant pathways as determined by pathway-enrichment analysis.

**Pathway**	**Total**	**Hits**	**Raw *p***	**Holm *p***	**Impact**
Glycerophospholipid metabolism	36	4	0.000	0.001	0.204
Glycerolipid metabolism	16	4	0.001	0.017	0.154
Caffeine metabolism	10	2	0.001	0.019	0.692
Sphingolipid metabolism	21	1	0.002	0.026	0.014

*Total corresponds to the total number of compounds in the pathway*.

*Hits correspond to the actually matched number from uploaded data*.

*Raw p is the original p-value calculated from the enrichment analysis*.

*Holm p is the p-value adjusted by the Holm-Bonferroni method*.

## Discussion

In this study, we analyzed the comprehensive metabolome of ionic and lipid molecules in urine samples from AD patients and cognitively normal, age- and sex-matched controls. This allowed us to develop a metabolic profile of AD urine. Five molecules had a significantly higher concentration, and 21 had a significantly lower concentration, in AD urine compared with control urine. These 26 molecules effectively discriminated the AD group from the control group in the OPLS-DA model. Results of pathway searches with the KEGG database, and pathway enrichment and topology analysis with Metaboanalyst software, suggested alterations in molecules relevant to pathways of glycerolipid and glycerophospholipid metabolism, thermogenesis, and caffeine metabolism in AD urine.

Metabolomics studies of blood and brains of AD patients found altered levels of glycerolipids and glycerophospholipids (7, 33–38). G3P initiates the first step in the biosynthesis of glycerolipids and glycerophospholipids (Supplementary Figure 3) (39). G3P and FA generate glycerolipids, including lysophosphatidic acid, phosphatidic acid, monoacylglycerols, diacylglycerols (DG), and triacylglycerols (TG). Hydrolysis of these acylglycerols results in FA release. PC is generated from DG, and PE from CDP-choline and CDP-ethanolamine (Supplementary Figure 3).

In the present study, G3P and ethanolamine phosphate were significantly lower in AD urine in bivariate analysis and contributed to the discriminant model with a high rank, and choline levels tended to be lower in AD urine (*p* = 0.06, see the ‘Processed data’ sheet of the Supplementary Datasets). Furthermore, the total amount of FA and DG was significantly lower in AD urine (Supplementary Table 6), and 5 FA, 2 TG, and 1 DG molecules were also significantly lower in AD urine, implying dysfunction in glycerolipid and glycerophospholipid metabolism in AD patients.

Previous studies have reported on changes in PC levels in brains and blood of AD patients. Specifically, lower levels of PCs with polyunsaturated fatty acid (PUFA) side chains were repeatedly observed (40–42). Essential PUFAs such as docosahexaenoic acid (DHA, 22:6) and arachidonic acid (AA, 20:4) provide structural functionality as phospholipid components in bilayer membranes (42). Meanwhile, Huo et al. reported that higher levels of some PC species containing saturated FA side chains are associated with future cognitive impairment (33). González-Domínguez et al. reported that PCs containing saturated and short-chain FAs in the serum of AD patients tended to increase, while PCs with PUFA side chains tended to decrease (43). Mapstone et al. reported that lower PC levels predict future cognitive impairment most acutely, but do not adequately discriminate between MCI/AD patients and cognitively normal controls (41). No significant changes were observed in most of the PC species found in the present study which contained saturated FA or non-essential PUFA side chains. This might be due to the cross-sectional design of our study to compare AD patients with older adults with normal cognitive function.

Levels of plasmalogen-type PE (ethanolamineplasmalogen, PlsPE), i.e., alkenyl PE, are reportedly reduced in brains and blood of AD patients (44). In the present study, levels of two PlsPEs were higher in AD urine, and the alkenyl side chain of each PlsPE was AA. To our knowledge, two studies have analyzed PlsPE in the blood of AD patients, including differences in their acyl chains (45, 46). Yamashita et al. reported that levels of plasma PlsPE containing DHA were significantly lower in AD patients, but no significant difference was observed in levels of PlsPEs containing other types of unsaturated fatty acids, including AA (45). On the other hand, Goodenowe et al. reported reductions of both PlsPEs that contain DHA and AA (46). Further analysis of differences in the dynamics of PlsPEs in AD patients based on their FA components, as well as the relationship between the amount of phospholipids discharged into urine and the amount of phospholipids in the blood, is warranted.

Acylcarnitines (AcCas) are made from FAs esterified to carnitine molecules, and are produced on the outer surface of the mitochondrial membrane by carnitine palmitoyl transferase 1 to facilitate the transport of long chain FAs across the mitochondrial membrane for breakdown by β-oxidation (47). TG, FA, and AcCa are essential metabolites in lipid metabolism, energy homeostasis, and thermogenesis (39). In the present analysis, both the total amount of FA and AcCa molecules identified were significantly lower, and TGs tended to be lower, in AD urine (Supplementary Figure 3 and Supplementary Table 5). Levels of AcCas in the plasma or serum of AD patients or individuals with cognitive impairment have been examined in cross sectional settings, with inconsistent results. Two studies reported lower levels of some AcCas in AD patients compared to healthy elderly individuals (48, 49), while two other studies reported a significant increase in certain AcCas in AD patients (50, 51). In prospective study settings, lower levels of some AcCas were found to predict future cognitive impairment or AD (33, 41). Since AcCas are not easily reabsorbed in the renal tubules (52), urinary AcCa concentrations reflect AcCa concentrations in the blood. On the other hand, levels of AcCas are affected by diet (52, 53). Studies that take into account subject body size (e.g., BMI) and food intake will be needed in the future.

Caffeine is a purine alkaloid that is consumed mainly from coffee and tea. Paraxanthine is the preferential product of caffeine metabolism in humans (54). In epidemiological studies, data on the protective effects of caffeine on cognitive decline and dementia have been mixed. A meta-analysis of eight prospective studies found no significant association between the amount of caffeine intake and AD risk (55), whereas another systematic review reported a positive association between caffeine intake and the risk of dementia/cognitive decline (56). A more recent meta-analysis found that mild coffee consumption is linked to a reduced risk of cognitive deficits (57). Therefore, caffeine intake may be associated with a decreased risk of dementia. However, we did not assess the amount of caffeine consumption in the present study.

In the present study, taurine and leucine were significantly lower in AD urine. Taurine is the most abundant sulfur-containing amino acid, with many functions in the nervous system (58). Lower serum levels of taurine are associated with a higher risk of dementia (41, 59–62). An association between increased levels of leucine, an essential amino acid, and a lower risk of dementia was reported in a recent study (63). Circulating levels of these amino acids are largely determined by dietary intake, and reduced levels might indicate reduced nutritional intake in AD patients. Weight loss later in life is known to be associated with a higher risk of dementia (64); thus, these metabolites may serve as potential markers for AD.

Given the diversity of lipid species, currently available methods can only analyze a subset of the full lipidome. Here, we took advantage of LC-FTMS for lipidomic analysis. LC-FTMS enables high mass resolution and highly selective detection of lipid species (27, 32, 65). To our knowledge, few reports have touched on the molecules identified in this study, such as FAs with very long chains (C27–C36) and sphingolipids with an atypical sphingoid base.

FAs in biological systems contain typically between 14 and 24 carbon atoms (66), and FAs with carbon chains of 26 or longer are often classified as ultra-long chain FAs (ULCFAs) (67). In the present study, saturated ULCFAs were observed and their levels were lower in AD patients. Saturated ULCFAs are found in small amounts in the brain, but are present in large amounts in skin and meibomian glands (67, 68). Significant amounts of wax esters, which are esters of FA and fatty alcohols, are contained in meibomian gland secretions and sebum (69–71). Depending on the analysis method, including that used in the present study, it is difficult to discriminate isomers between FA and wax esters. To our knowledge, no other study has reported on saturated ULCFAs in urine, and we cannot exclude the possibility that most (but not all) saturated ULCFAs identified in the present study are wax esters. Further investigation on urine metabolomics using a similar method is warranted.

Sphingolipids are characterized by the presence of a common sphingoid backbone structure, such as sphingosine (d18:1), sphinganine or dihydrosphingosine (d18:0), and phytosphingosine or 4-hydroxysphinganine (t18:0) as sphingoid bases in humans (72). The sphingoid bases of m19:0, m17:1, and t17:1 identified in Hex1Cer, Cer, and CerP were atypical. To our knowledge, this is the first report to identify these species in urine.

Hex1Cer is a cerebroside that includes glucosylceramide and galactosylceramide, a member of a group of glycosphingolipids that are highly abundant in the vertebrate brain (73). In the present study, the level of Hex1Cer (m19:0_21:2) was significantly higher and the total amount of Hex1Cer tended to higher, in AD urine. In mammals, glucosylceramide and galactosylceramide are catabolized by glucocerebrosidase and galactocerebrosidase, respectively, with the assistance of saposin A and saposin C, respectively, (74). Interestingly, we previously reported that levels of prosaposin (PSAP), the precursor of saposins, were significantly lower in AD urine relative to control urine (30).

The influence of medication on our present results cannot be ruled out. Donepezil, a representative drug used to treat AD, may potentially interact with drugs metabolized via CYP1A2-, CYP2D6-, and CYP3A4-related enzymes (75), and CYP1A2 is a major enzyme catalyzing the metabolism of caffeine. Wan et al. compared blood metabolites of AD patients treated with donepezil for at least 3 months and reported that blood G3P levels were significantly lower in the donepezil responsive group, although no difference was found in blood G3P levels between individuals newly diagnosed with AD and healthy controls (76). In the AD group of the present study, an average of 4 years had passed since diagnosis and 10/18 participants were being treated with donepezil.

This study has some limitations worth noting. Urinary metabolites and lipidomes are highly influenced intra- and inter-individually by factors such as diet, alcohol consumption, exercise, drugs, sex, and age. The small sample size and use of spot urine samples without any restrictions prior to urine collection are primary limitations of this study.

Second, BMI differed between the AD and control groups. The AD group had significantly lower BMI, although 7 of 18 participants had missing BMI data. Reduced appetite and weight loss are common symptoms of AD (64, 77) and may explain the lower urinary levels of AcCas, FAs, TGs, taurine, and leucine observed in the study, as well the increase in PC (18:0_20:3). The acyl group of PC (18:0_20:3) is presumably derived from stearic acid and mead acid. Mead acid is reported to be produced in the absence of essential FAs (78–80). Our results suggest that AD patients may have reduced food intake, although we cannot rule out the possibility that intracellular energy metabolism might be impaired in these patients.

Third, there was limited clinical information on our participants. The diagnosis of AD was based on NIA-AA criteria, whereas their cognitive function was determined only by the MMSE; no other cognitive function tests (e.g., CDR) were employed. Based on their MMSE scores, however, we estimated that they had a relatively wide range of dementia severity, with CDRs ranging from 0.5 to 2 (mainly no MCI).

Finally, the present study used a cross-sectional, case-control design, which did not allow us to evaluate causality. Prospectively designed studies that include detailed information on cognitive changes and lifestyle habits will be necessary in the future.

In conclusion, we demonstrated differences in the urinary metabolome between AD patients and cognitively normal individuals, and identified a panel of molecules that can discriminate between the two groups. Urinary metabolite profiles of AD mainly suggested alterations in glycerolipid and FA metabolic pathways. Advances in technology are expected to reveal more metabolites in urine, and urinary biomarker discovery will contribute to early detection and an understanding of AD pathogenesis.

## Data Availability Statement

The original contributions presented in the study are included in the article/Supplementary Material, further inquiries can be directed to the corresponding author.

## Ethics Statement

The studies involving human participants were reviewed and approved by The Human Research Ethics Committee of Niigata University. Written informed consent for participation was not required for this study in accordance with the national legislation and the institutional requirements.

## Author Contributions

YW: conducted the study, collected the data, preformed the statistical analysis, interpreted the data, and wrote the manuscript. KKa, TT, and KKi: collected the data, interpreted the results, and revised the manuscript. TI: advised on the study design, supervised the data collection, and contributed to manuscript writing and revision. KN: conceptualized the study, supervised the data collection, advised on the statistical analysis, and critically revised the manuscript. All authors read and approved the final manuscript.

## Funding

Financial support was provided in part by JSPS KAKENHI Grant Numbers JP23249035 and JP15H04782 to KN, JP17K19799 and JP19K21581 to KN and YW, and JP16K09051 to YW, and a grant from the Japan Agency for Medical Research and Development (AMED) to TI (Grant Number 18dm0107143).

## Conflict of Interest

The authors declare that the research was conducted in the absence of any commercial or financial relationships that could be construed as a potential conflict of interest.

## Publisher's Note

All claims expressed in this article are solely those of the authors and do not necessarily represent those of their affiliated organizations, or those of the publisher, the editors and the reviewers. Any product that may be evaluated in this article, or claim that may be made by its manufacturer, is not guaranteed or endorsed by the publisher.
