# Supplementary material for: Alterations in Glycerolipid and Fatty Acid Metabolic Pathways in Alzheimer's Disease Identified by Urinary Metabolic Profiling: A Pilot Study
Source: Front Neurol. 2021 Oct 27;12:719159. doi: 10.3389/fneur.2021.719159 (PMC8578168; doi:10.3389/fneur.2021.719159)
Supplement: Supplementary file 1 [file Data_Sheet_1.PDF]

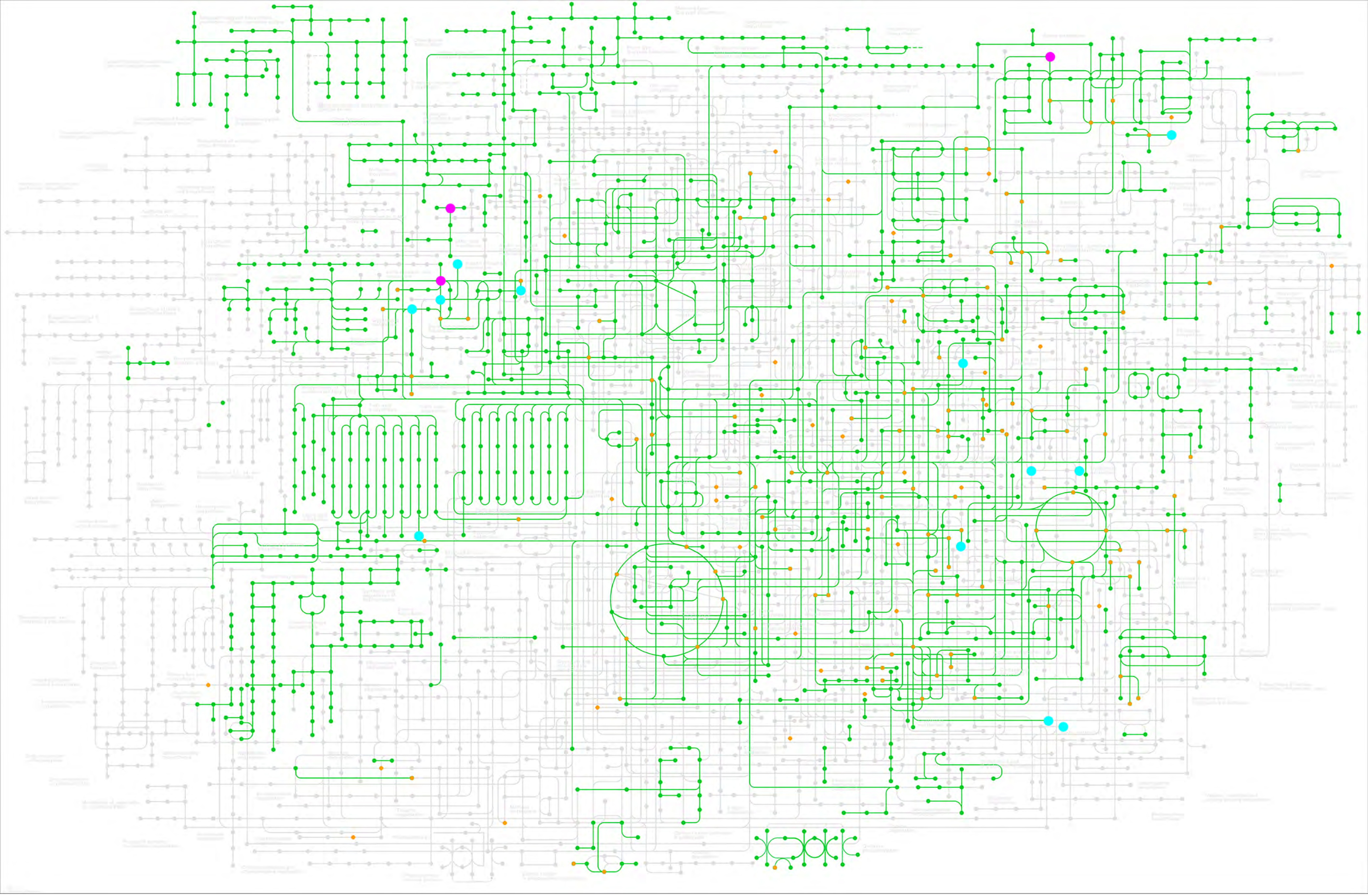

Supplementary Fig. 1A. KEGG pathway map of global metabolic map. Circles represent compounds (metabolites).

Red and cyan circles represent significantly increased and decreased metabolites in AD urine, respectively.

Orange circles are compounds that were found in this study without a statistically significant difference. Green circles and lines represent pathways identified in humans.

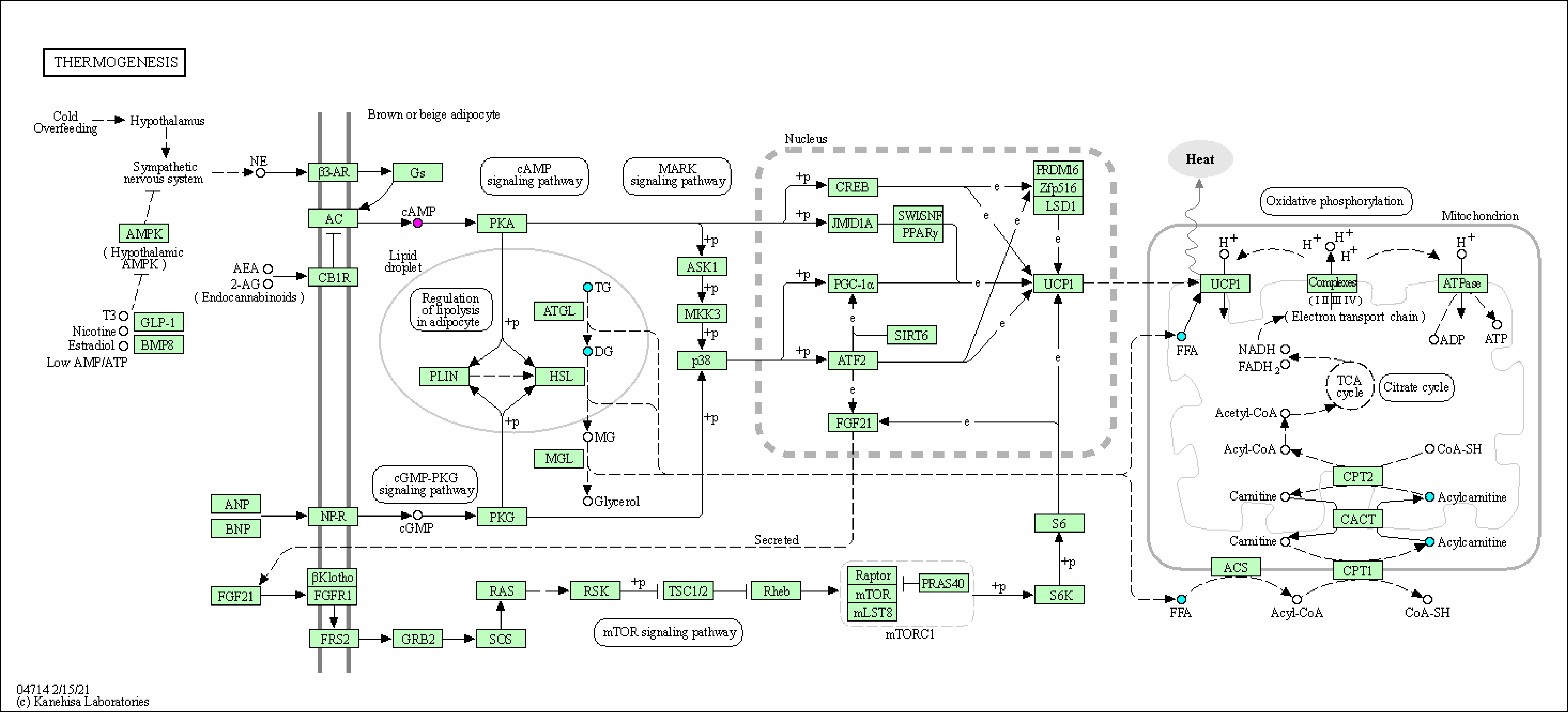

Supplementary Fig. 1B. KEGG pathway map of thermogenesis. Circles represent compounds (metabolites), and rectangles represent genes. Red and cyan circles represent significantly increased and decreased metabolites in AD urine, respectively. Green rectangles represent genes identified in humans.

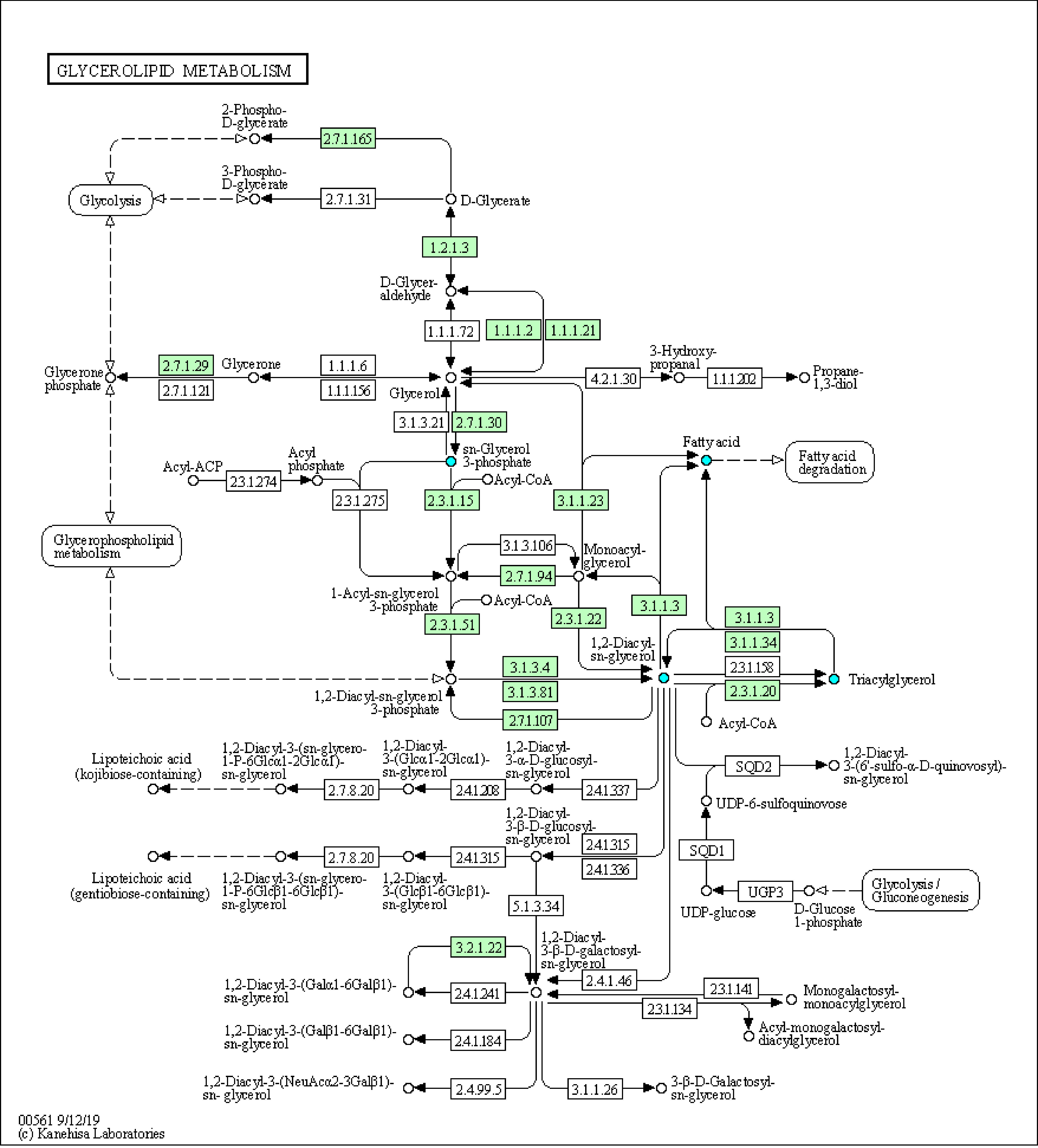

Supplementary Fig. 1C. KEGG pathway map of glycerolipid metabolism. Circles represent compounds (metabolites), and rectangles represent genes. Red and cyan circles represent significantly increased and decreased metabolites in AD urine, respectively. Green rectangles represent genes identified in humans.

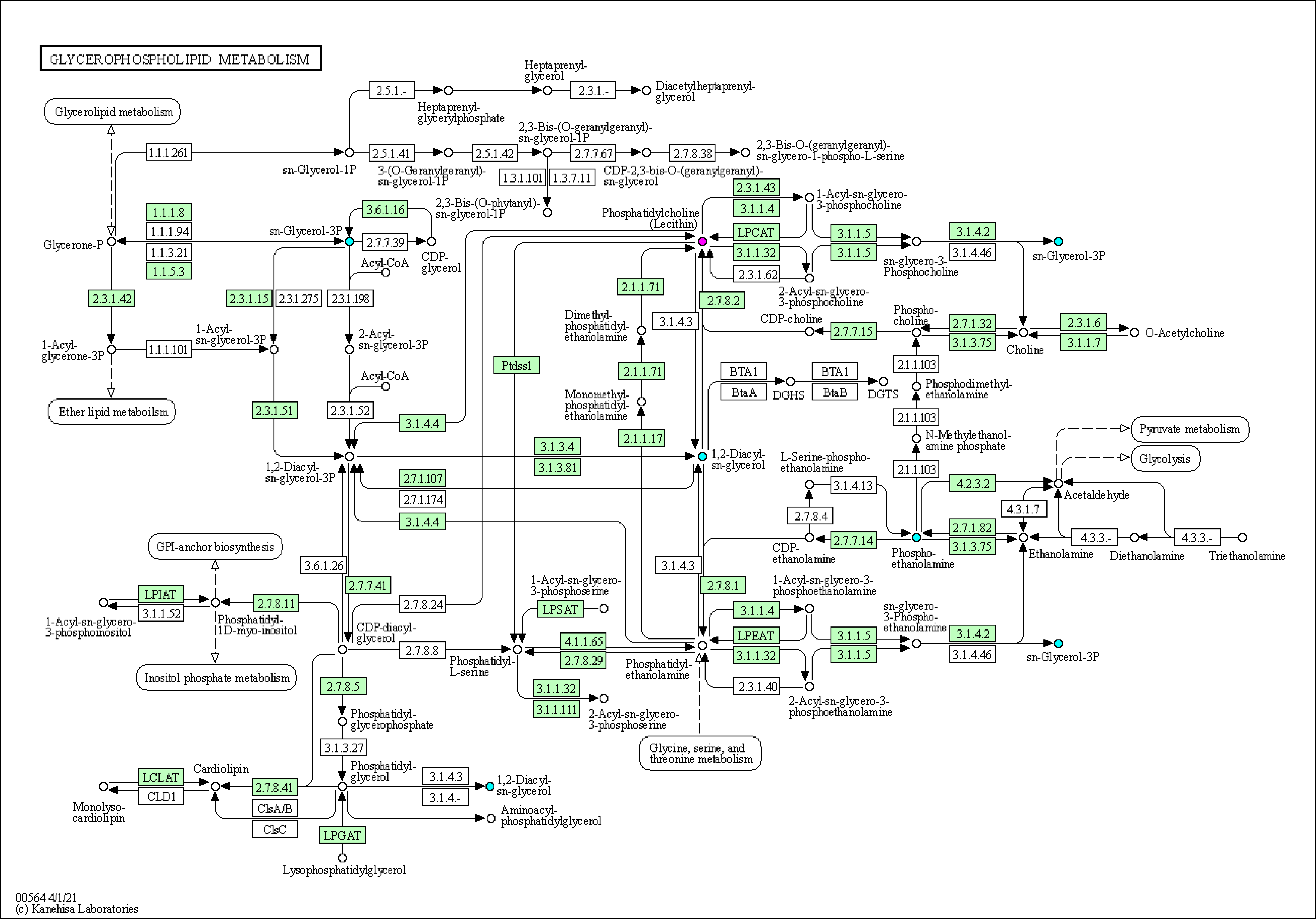

Supplementary Fig. 1D. KEGG pathway map of glycerophospholipid metabolism. Circles represent compounds (metabolites), and rectangles represent genes. Red and cyan circles represent significantly increased and decreased metabolites in AD urine, respectively. Green rectangles represent genes identified in humans.

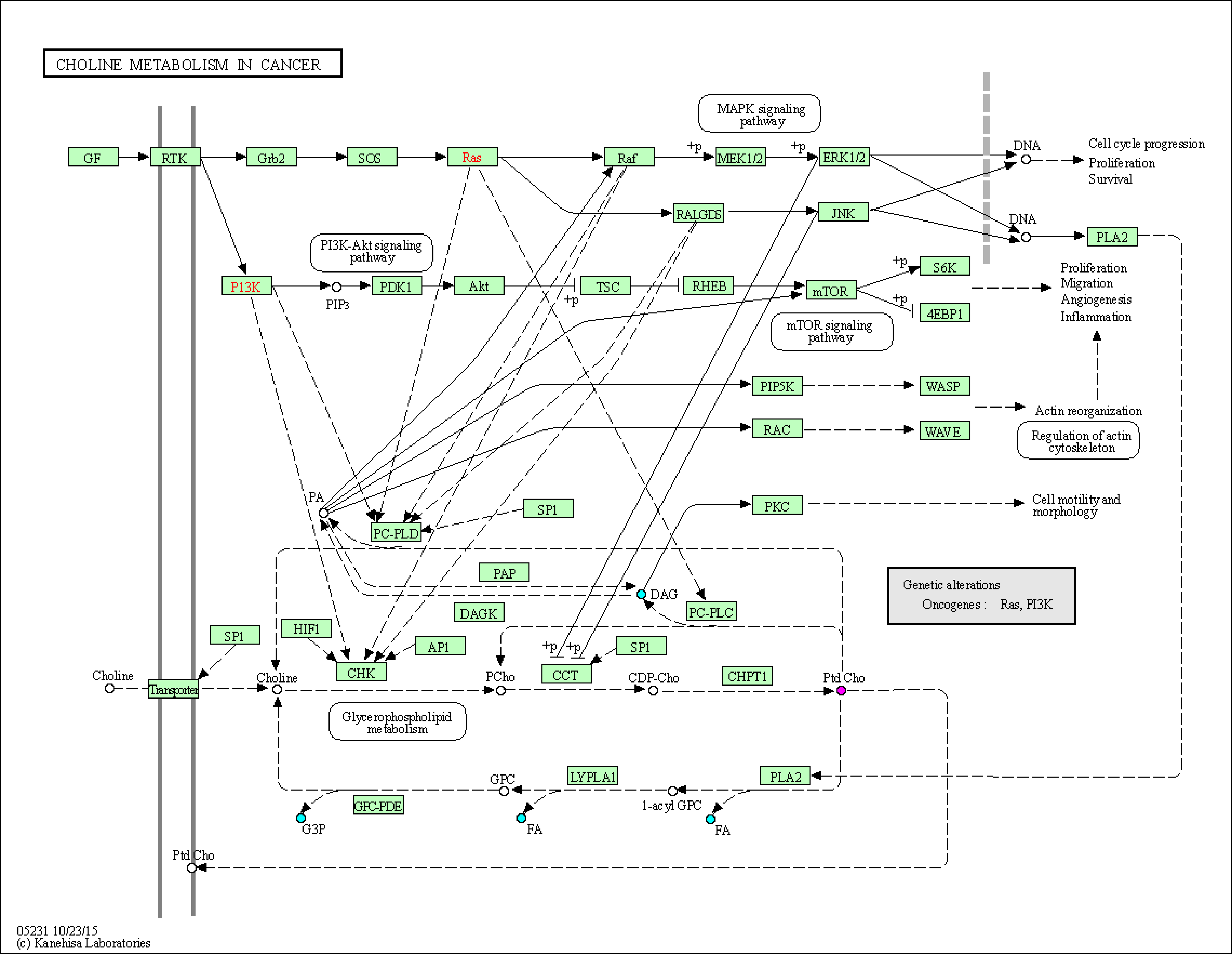

Supplementary Fig. 1E. KEGG pathway map of Choline metabolism in cancer. Circles represent compounds (metabolites), and rectangles represent genes. Red and cyan circles represent significantly increased and decreased metabolites in AD urine, respectively. Green rectangles represent genes identified in humans.

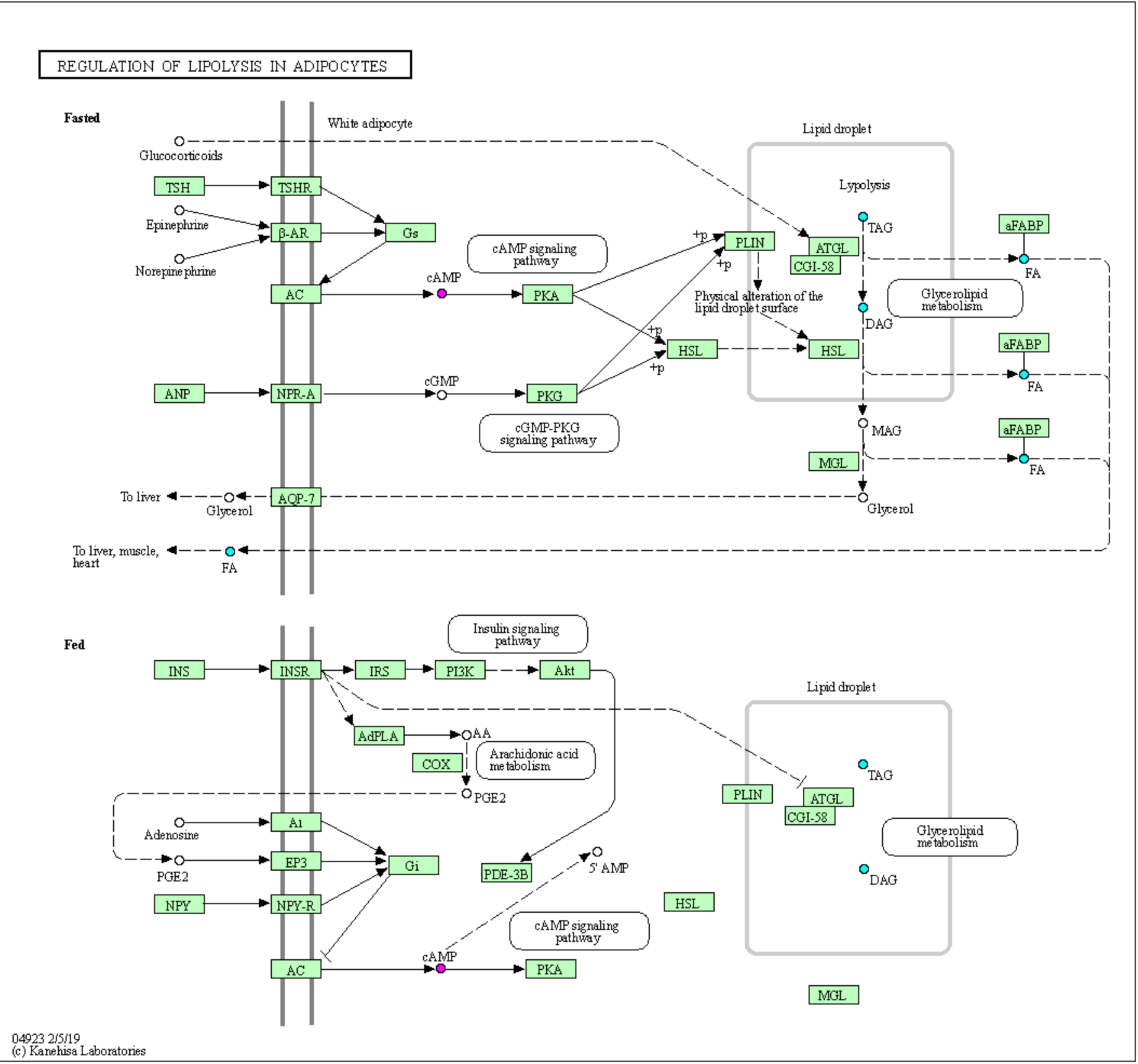

Supplementary Fig. 1F. KEGG pathway map of regulation of lipolysis in adipocytes. Circles represents com-pounds (metabolites), and rectangles represent genes. Red and cyan circle represent significantly increased and decreased metabolites in the AD urine, respectively. Green rectangles represent genes identified in human.
