# Supplementary material for: Alterations in Glycerolipid and Fatty Acid Metabolic Pathways in Alzheimer's Disease Identified by Urinary Metabolic Profiling: A Pilot Study
Source: Front Neurol. 2021 Oct 27;12:719159. doi: 10.3389/fneur.2021.719159 (PMC8578168; doi:10.3389/fneur.2021.719159)
Supplement: Supplementary file 2 [file Data_Sheet_2.pdf]

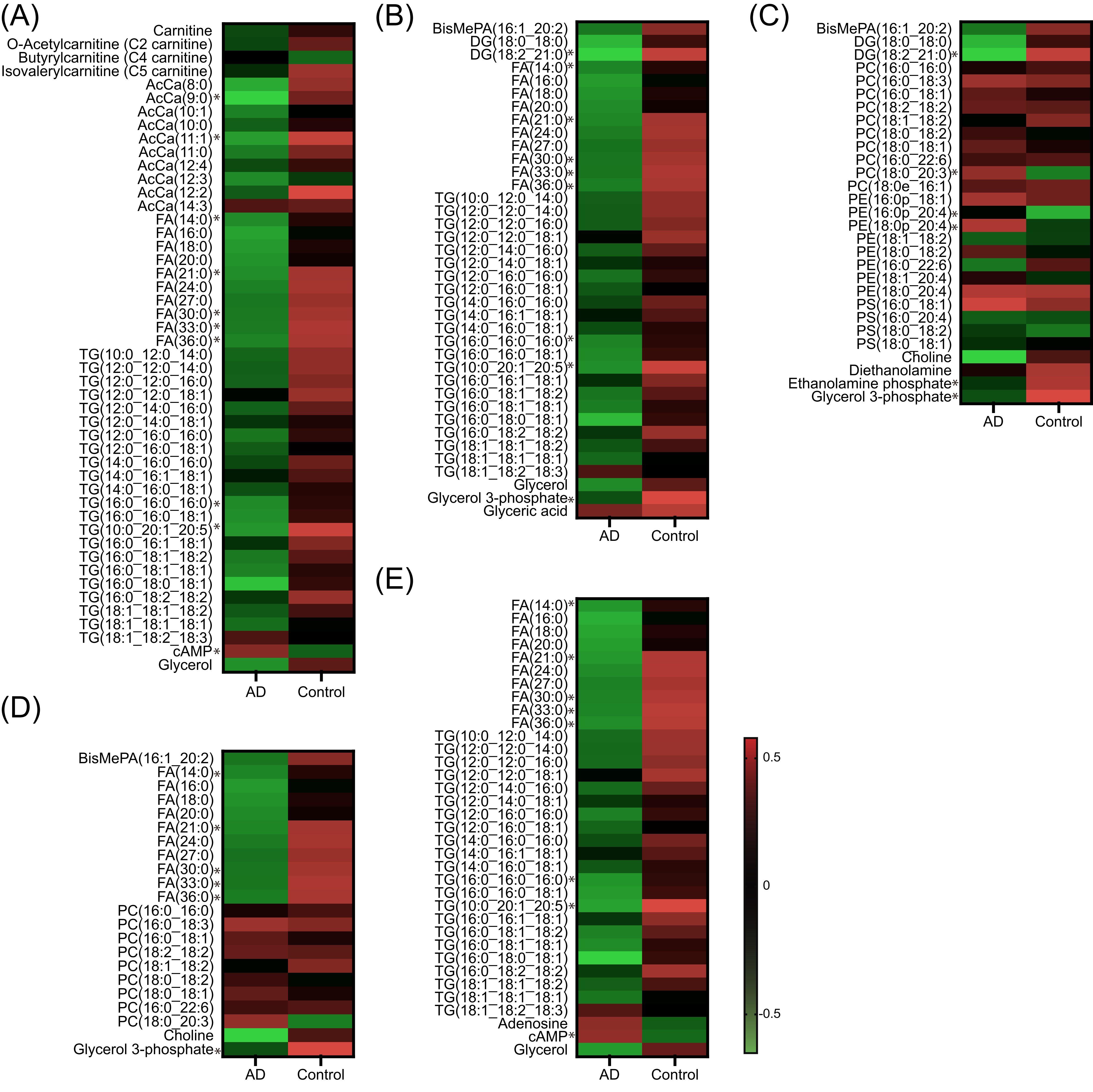

Supplementary Figure S2. Heatmap representation of all metabolites identified in each pathway shown in Table 2 (except for hsa01100). Pathways of (A) thermogenesis, (B) glycerolipid metabolism, (C) glycerophospholipid metabolism, (D) choline metabolism in cancer, and (E) regulation of lipolysis in adipocytes. Asterisks indicate molecules that significantly differed between AD and control groups.
