# Supplementary material for: Alterations in Glycerolipid and Fatty Acid Metabolic Pathways in Alzheimer's Disease Identified by Urinary Metabolic Profiling: A Pilot Study
Source: Front Neurol. 2021 Oct 27;12:719159. doi: 10.3389/fneur.2021.719159 (PMC8578168; doi:10.3389/fneur.2021.719159)
Supplement: Supplementary file 3 [file Data_Sheet_3.pdf]

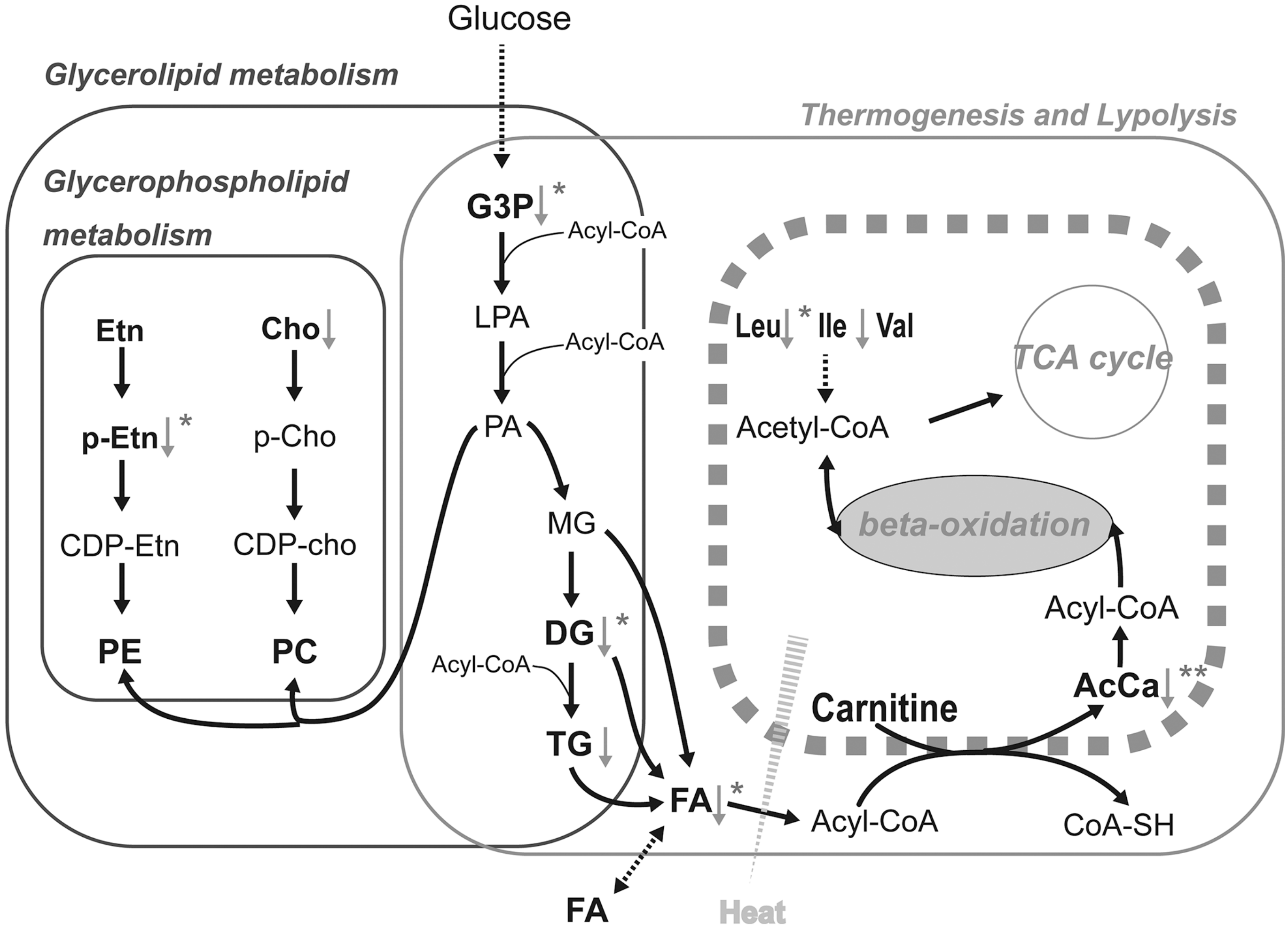

Supplementary Figure.S3. Schematic of glycerolipid metabolism, glycerophospholipid metabolism, thermogenesis, and lipolysis pathways. Molecules in bold were identified in the study. The downward arrow after the molecule name indicates that the concentration observed in AD urine was lower than that in control urine (No asterisk, $p<0.1$, * $p<0.05$, and ** $p<0.01$).
