# Supplementary material for: Alterations in Glycerolipid and Fatty Acid Metabolic Pathways in Alzheimer's Disease Identified by Urinary Metabolic Profiling: A Pilot Study
Source: Front Neurol. 2021 Oct 27;12:719159. doi: 10.3389/fneur.2021.719159 (PMC8578168; doi:10.3389/fneur.2021.719159)
Supplement: Supplementary file 4 [file Data_Sheet_4.pdf]

Supplementary Fig. S4.

Peak areas (raw data) of representative compounds including the blank sample (BK). A1-A18 are samples from AD patients and C1-C18 are samples from controls.

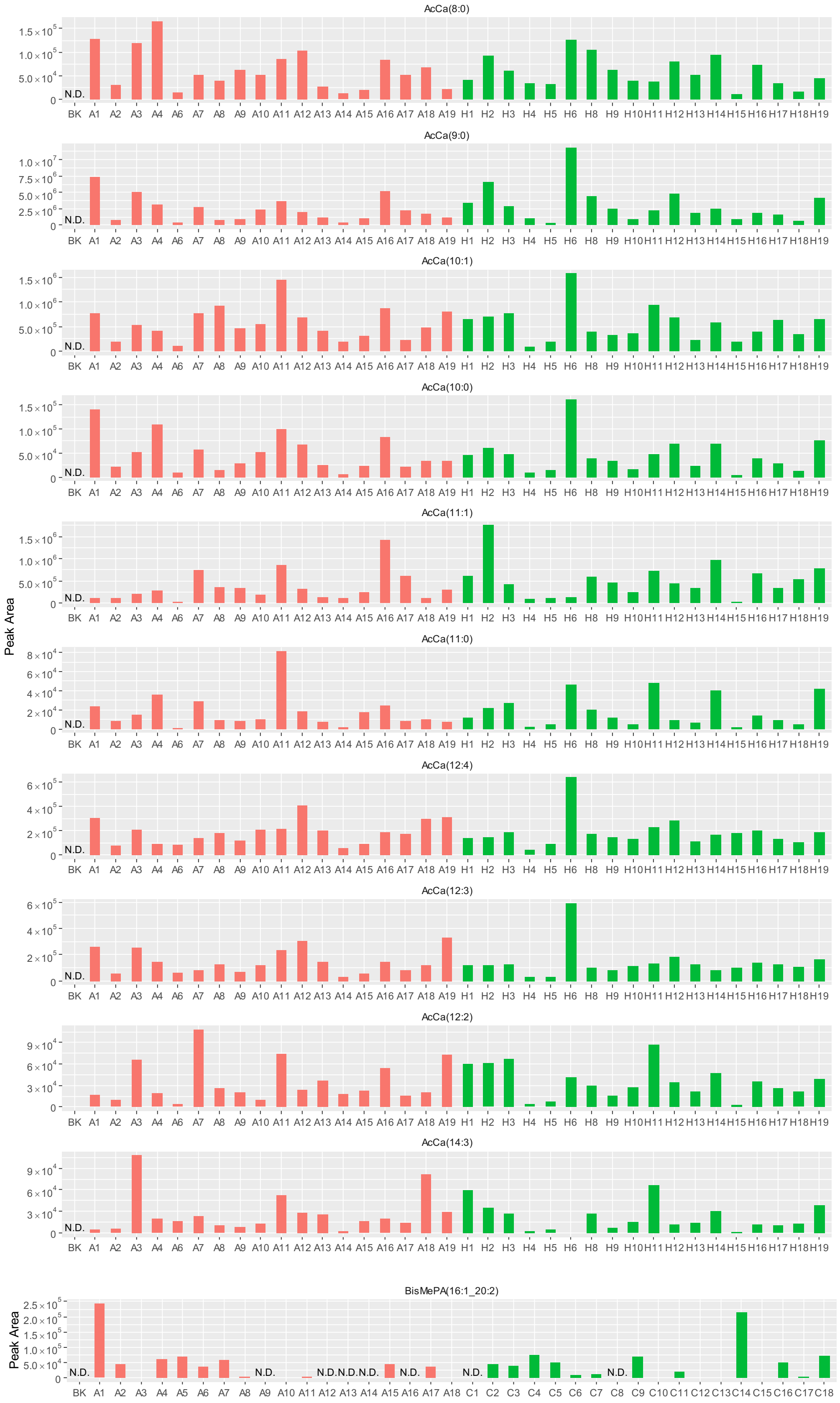

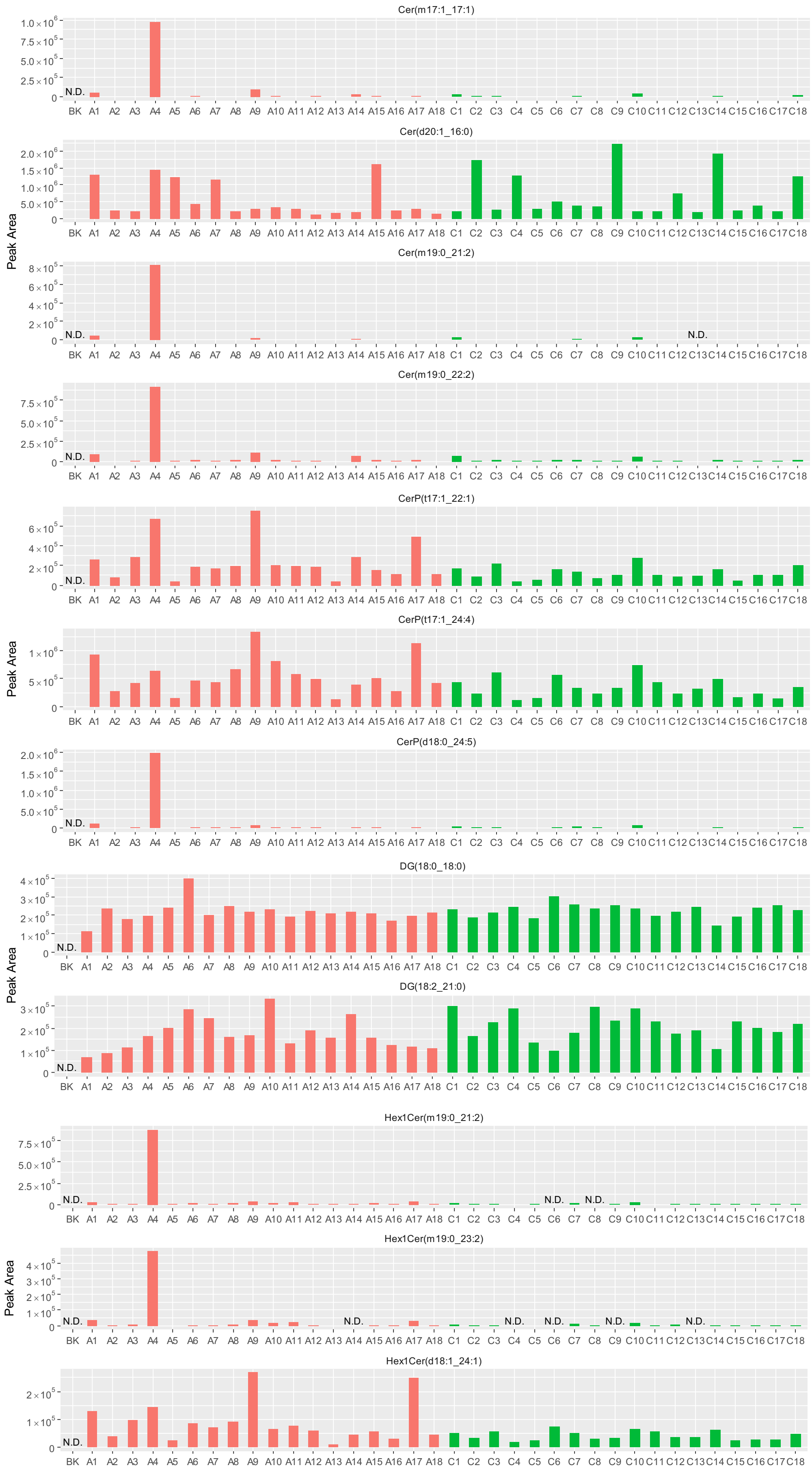
Cer(m17:1_17:1)
Cer(d20:1_16:0)
Cer(m19:0_21:2)
Cer(m19:0_22:2)
CerP(t17:1_22:1)
CerP(t17:1_24:4)
CerP(d18:0_24:5)
DG(18:0_18:0)
DG(18:2_21:0)
Hex1Cer(m19:0_21:2)
Hex1Cer(m19:0_23:2)
Hex1Cer(d18:1_24:1)
Peak Area
N.D.
BK A1 A2 A3 A4 A5 A6 A7 A8 A9 A10 A11 A12 A13 A14 A15 A16 A17 A18 C1 C2 C3 C4 C5 C6 C7 C8 C9 C10 C11 C12 C13 C14 C15 C16 C17 C18

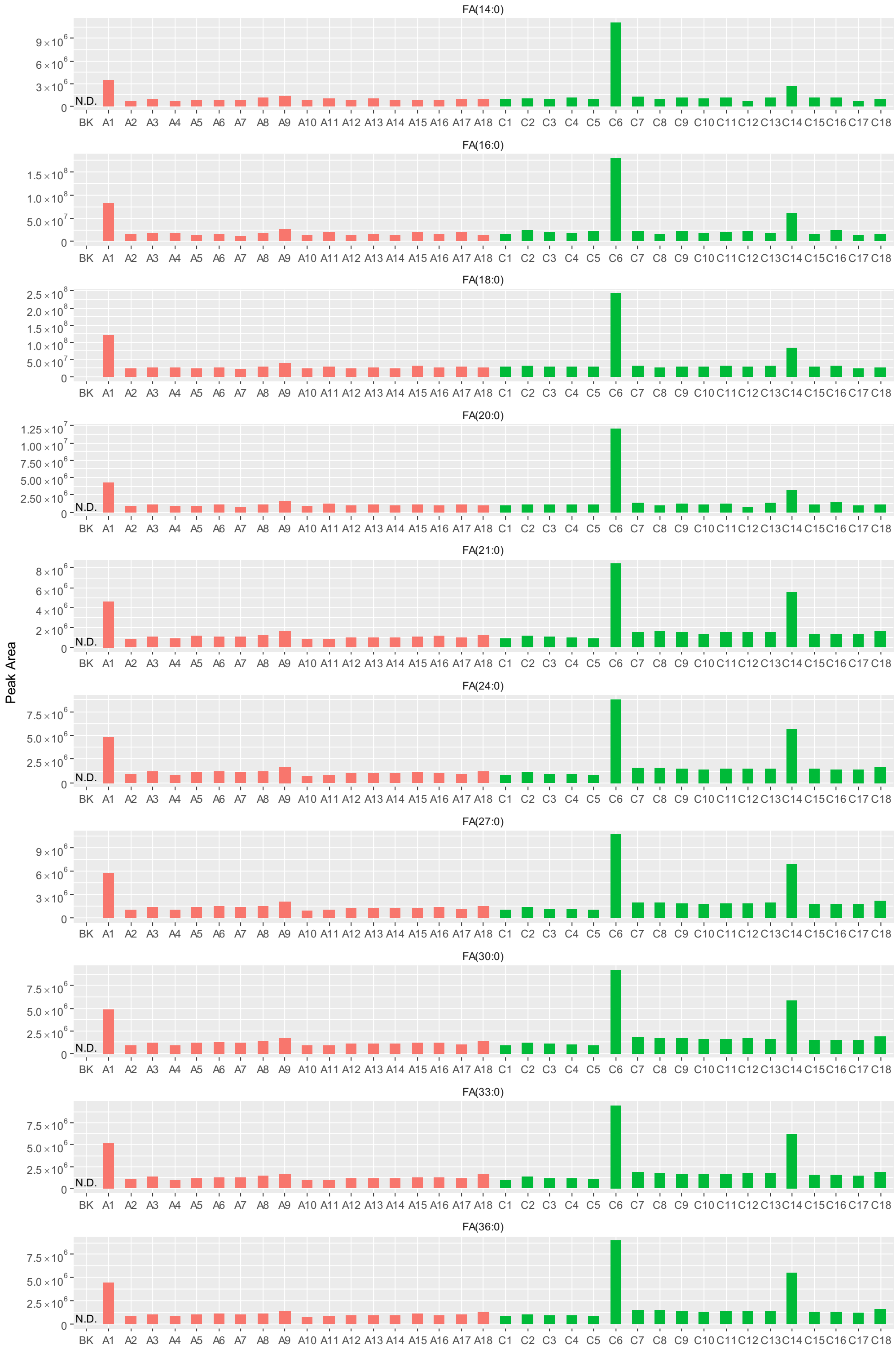
FA(14:0)
FA(16:0)
FA(18:0)
FA(20:0)
FA(21:0)
FA(24:0)
FA(27:0)
FA(30:0)
FA(33:0)
FA(36:0)
Peak Area
N.D.
BK A1 A2 A3 A4 A5 A6 A7 A8 A9 A10 A11 A12 A13 A14 A15 A16 A17 A18 C1 C2 C3 C4 C5 C6 C7 C8 C9 C10 C11 C12 C13 C14 C15 C16 C17 C18

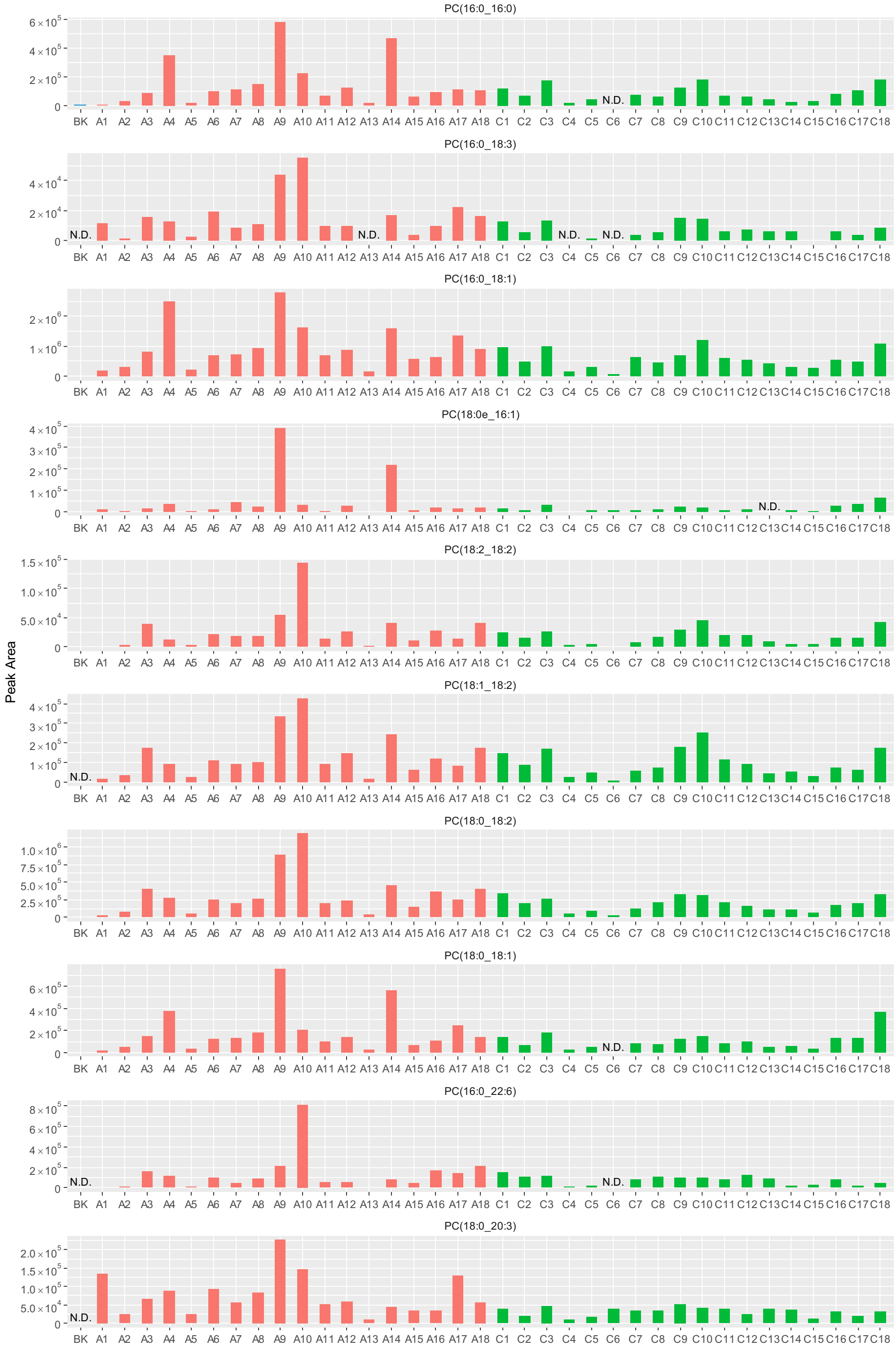
PC(16:0_16:0)
PC(16:0_18:3)
PC(16:0_18:1)
PC(18:0e_16:1)
PC(18:2_18:2)
PC(18:1_18:2)
PC(18:0_18:2)
PC(18:0_18:1)
PC(16:0_22:6)
PC(18:0_20:3)
Peak Area
N.D.
BK A1 A2 A3 A4 A5 A6 A7 A8 A9 A10 A11 A12 A13 A14 A15 A16 A17 A18 C1 C2 C3 C4 C5 C6 C7 C8 C9 C10 C11 C12 C13 C14 C15 C16 C17 C18

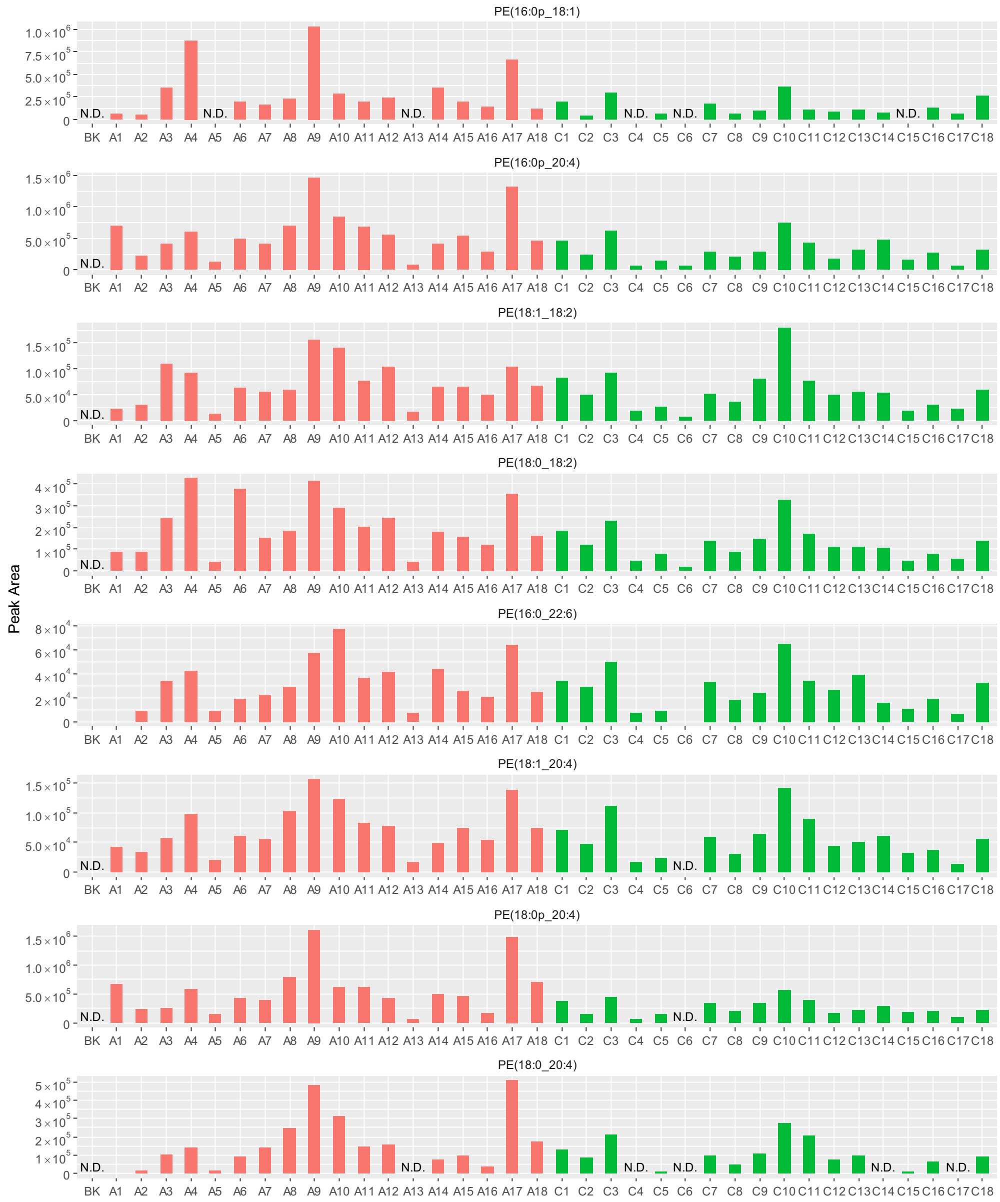
PE(16:0p_18:1)
PE(16:0p_20:4)
PE(18:1_18:2)
PE(18:0_18:2)
PE(16:0_22:6)
PE(18:1_20:4)
PE(18:0p_20:4)
PE(18:0_20:4)
Peak Area
N.D.
BK A1 A2 A3 A4 A5 A6 A7 A8 A9 A10 A11 A12 A13 A14 A15 A16 A17 A18 C1 C2 C3 C4 C5 C6 C7 C8 C9 C10 C11 C12 C13 C14 C15 C16 C17 C18

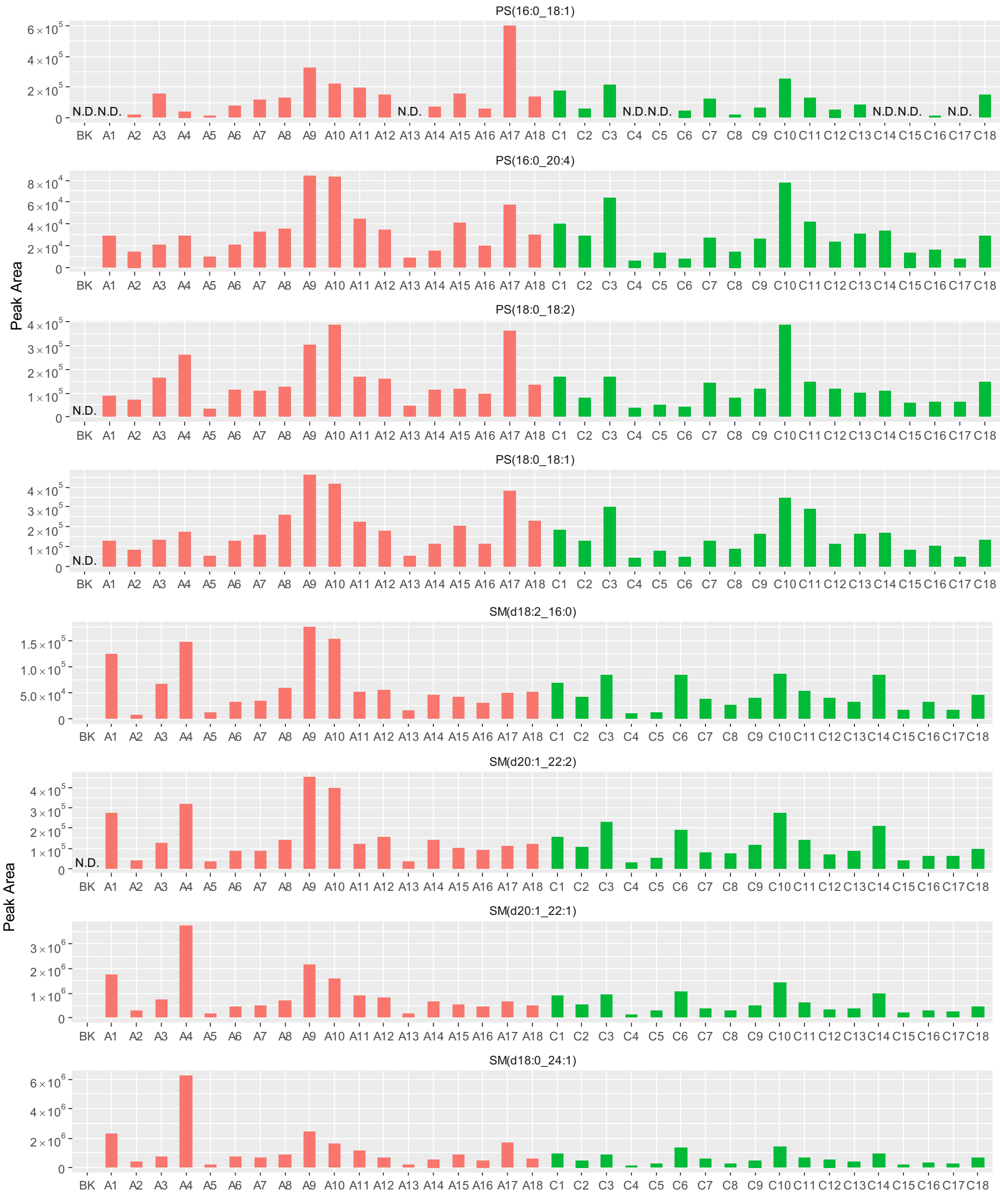
PS(16:0_18:1)
PS(16:0_20:4)
PS(18:0_18:2)
PS(18:0_18:1)
SM(d18:2_16:0)
SM(d20:1_22:2)
SM(d20:1_22:1)
SM(d18:0_24:1)
Peak Area
Peak Area
N.D.
BK A1 A2 A3 A4 A5 A6 A7 A8 A9 A10 A11 A12 A13 A14 A15 A16 A17 A18 C1 C2 C3 C4 C5 C6 C7 C8 C9 C10 C11 C12 C13 C14 C15 C16 C17 C18

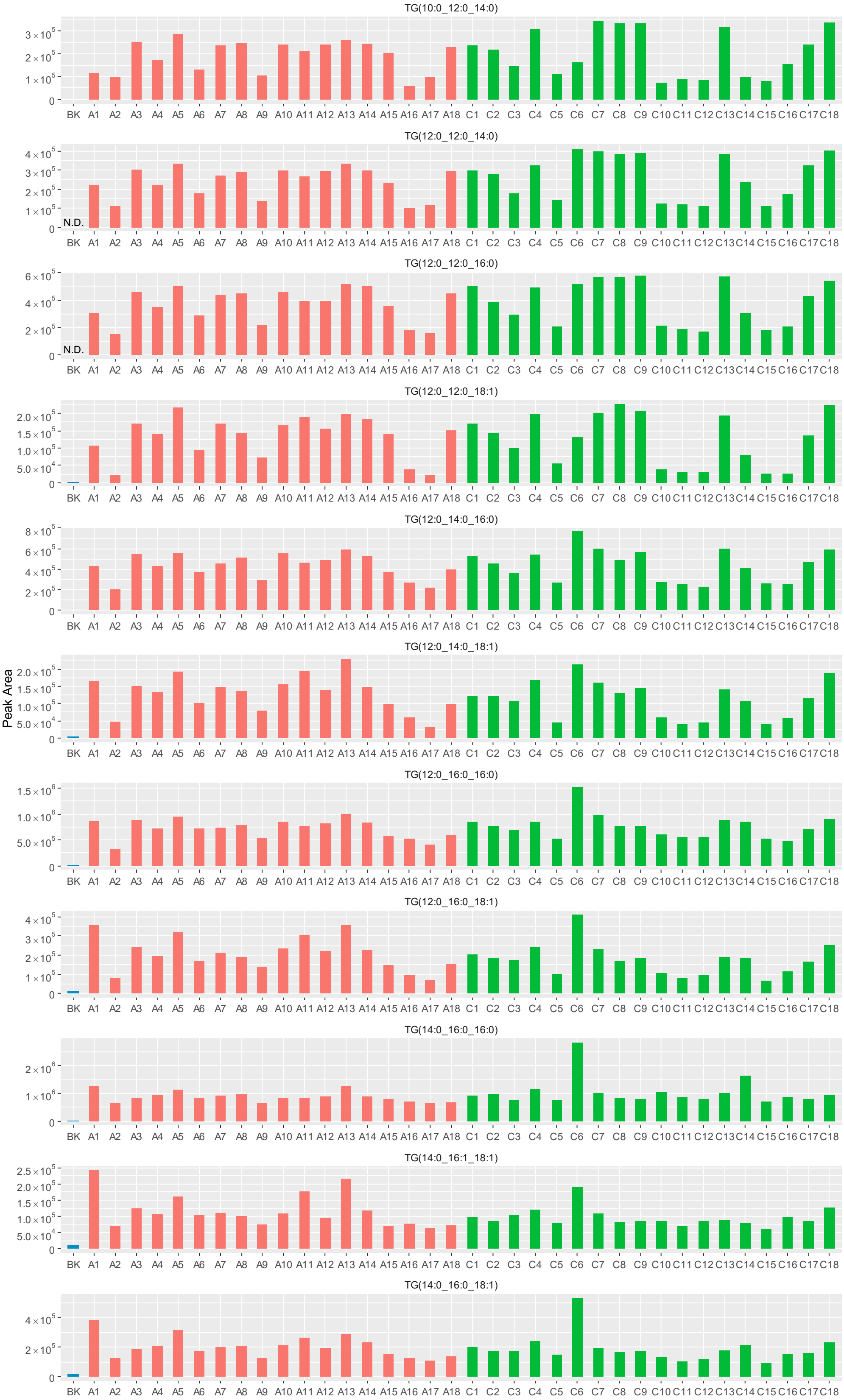

TG(10:0_12:0_14:0)
TG(12:0_12:0_14:0)
TG(12:0_12:0_16:0)
TG(12:0_12:0_18:1)
TG(12:0_14:0_16:0)
TG(12:0_14:0_18:1)
TG(12:0_16:0_16:0)
TG(12:0_16:0_18:1)
TG(14:0_16:0_16:0)
TG(14:0_16:1_18:1)
TG(14:0_16:0_18:1)
Peak Area
N.D.
BK A1 A2 A3 A4 A5 A6 A7 A8 A9 A10 A11 A12 A13 A14 A15 A16 A17 A18 C1 C2 C3 C4 C5 C6 C7 C8 C9 C10 C11 C12 C13 C14 C15 C16 C17 C18

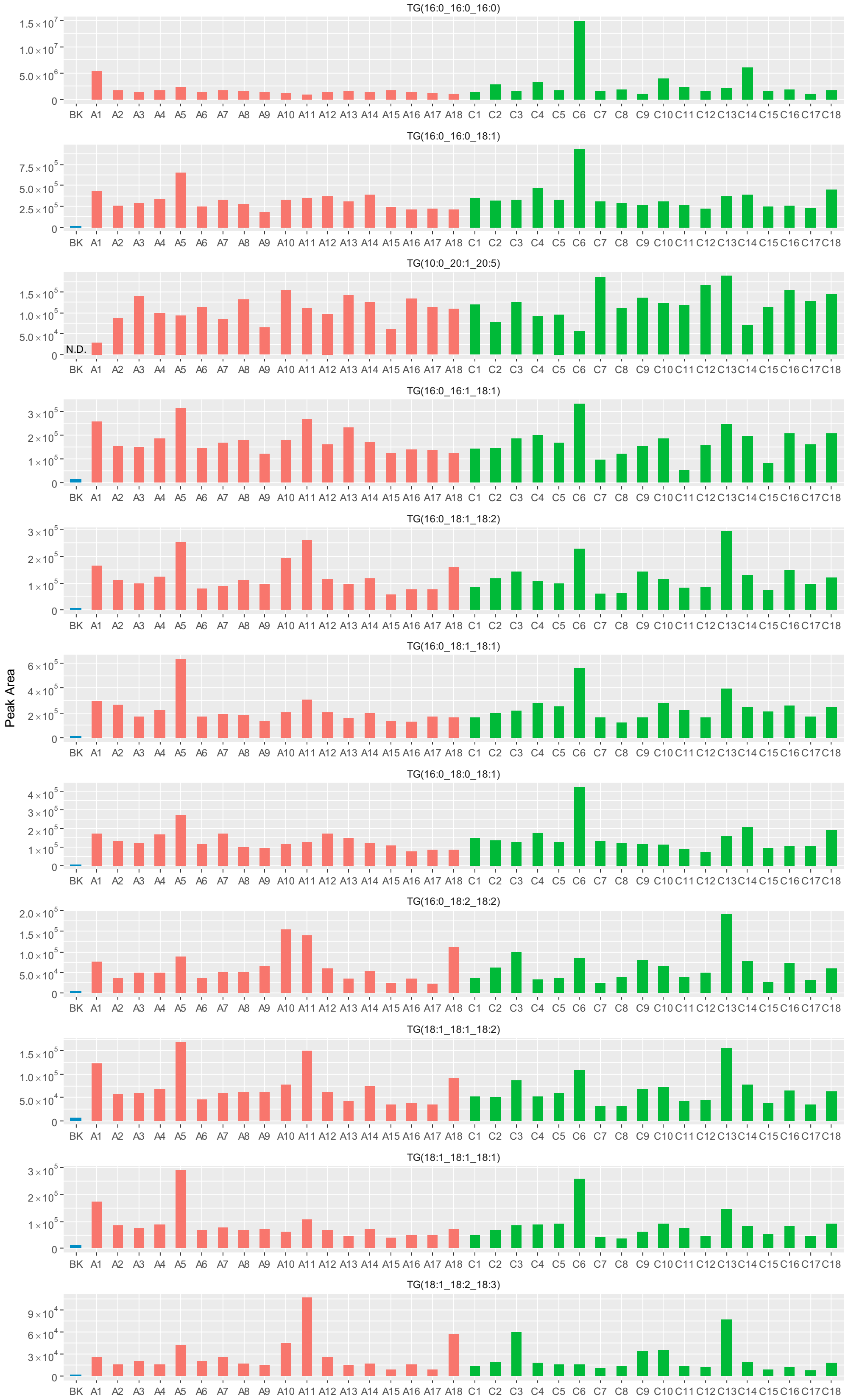
TG(16:0_16:0_16:0)
TG(16:0_16:0_18:1)
TG(10:0_20:1_20:5)
N.D.
TG(16:0_16:1_18:1)
TG(16:0_18:1_18:2)
TG(16:0_18:1_18:1)
TG(16:0_18:0_18:1)
TG(16:0_18:2_18:2)
TG(18:1_18:1_18:2)
TG(18:1_18:1_18:1)
TG(18:1_18:2_18:3)
Peak Area
BK A1 A2 A3 A4 A5 A6 A7 A8 A9 A10 A11 A12 A13 A14 A15 A16 A17 A18 C1 C2 C3 C4 C5 C6 C7 C8 C9 C10 C11 C12 C13 C14 C15 C16 C17 C18
