# Supplementary material for: Alterations in Glycerolipid and Fatty Acid Metabolic Pathways in Alzheimer's Disease Identified by Urinary Metabolic Profiling: A Pilot Study
Source: Front Neurol. 2021 Oct 27;12:719159. doi: 10.3389/fneur.2021.719159 (PMC8578168; doi:10.3389/fneur.2021.719159)
Supplement: Supplementary file 7 [file Table_3.DOCX]

Supplementary Methods

# Urine sample collection and laboratory test

The procedures for urine collection and storage are described elsewhere (1). Briefly, spot urine samples were obtained from participants. No restrictions on diet, drinking, or exercise were required prior to urine sampling. Collected urine samples were immediately stored at 4°C for 2 to 4 hours for AD patients and 3 to 8 hours for controls before centrifugation. Samples were centrifuged at 1,000 xg for 15 min, and the supernatant was aliquoted and stored at -80°C until use.

General urinalysis (urinary protein, urinary sugar, occult blood) was performed using urine test strips (Pretest 5bII, Wako, Japan, for AD urine; Hema-Combistix-long, Siemens Healthcare Japan, for control urine). Urinary creatinine was measured by latex immunological nephelometry using a SPOTCHEM D-01 analyzer (SD-3810, Arkray Global Business, Inc. Japan).

# Metabolomics analysis

Analyses of ionic metabolites were performed at Human Metabolome Technologies Inc. (HMT, Tsuruoka, Japan) using CE-TOFMS.

Urine (20 μl) was dissolved in 20 μl of 1000 μM internal standard solution and 60 μl Mili-Q water. The sample was filtered centrifugally through a 5 kDa cutoff filter (Ultrafree MC PLHCC, HMT) at 9,100 × g at 4 °C for 60 min. The filtrate was diluted with MiliQ-water 5 times for the cation mode and 3 times for the anion mode.

CE-TOFMS was carried out using an Agilent CE-TOFMS system (Agilent Technologies, Santa Clara, California) as described previously (2–4), with slight modifications. Separation of metabolites was carried out on 50 μm i.d. x 80 cm fused-silica capillaries with a commercial electrophoresis buffer (H3301-1001 for cation analysis and H3302-1021 for anion analysis; HMT) as the electrolyte. Samples were inserted with a pressure injection of 50 mbar for 10 sec and 25 sec, for cation mode and anion mode, respectively. The applied voltage was set at 27 kV and capillary voltage of 4000 V and 3500 V for cation mode and anion mode, respectively. The spectrum was scanned from m/z 50 to 1,000, and peaks were extracted using MasterHands, automatic integration software (Keio University, Tsuruoka, Japan), to obtain peak information including m/z, peak area, and migration time (MT) (5). Signal peaks corresponding to isotopomers, adduct ions, and other product ions of known metabolites were excluded, and remaining peaks were annotated according to the HMT metabolite database based on their m/z values with MTs determined by CE-TOFMS. Each peak area was then corrected to a relative area value by the peak area of creatinine.

The quality control (QC) of the measurement was based on the relative standard deviation of the internal standard solution (L-methionine sulfone for cationic mode and D-camphor-10-sulfonic acid for anionic mode) added to the sample being within 10%.

# Lipidomics analysis

Lipidomics analysis was performed at Chemicals Evaluation and Research Institute, Japan (CERI, Saitama, Japan) using LC-FTMS (6,7).

Urine (200 μl) was added to 1,000 μl of ethanol and dried with a centrifugal evaporator. Methanol (500 μl) was added, and ultrasonic waves were irradiated for 1 minute. Chloroform (500 μl) was added, and the extract solution was shaken with a multi-shaker at 2,500 rpm for 5 min and then centrifuged at 4 °C and 12,000 × g for 5 minutes. After 900 μl of the supernatant fluid were dried with a centrifugal evaporator, 100 μl of 500 ng/ml phosphatidylcholine (PC) (16:0D31-18:1) in methanol were added and ultrasonic waves were applied for 5 minutes. Supernatant (85 μl) was collected and 85 μl of methanol were added; 20 μl of each sample solution were collected and mixed to prepare a QC sample for LC-FTMS analysis.

LC–FTMS analysis was performed using a Prominence UFLC XR system (Shimadzu, Tokyo, Japan) coupled with a LTQ Orbitrap XL equipped with a heated electrospray ionization interface (H‐ESI) (Thermo Fisher Scientific, Waltham, MA, USA). Separation of lipids was carried out on an L-column 2 ODS metal free column (2 mm i.d. × 50 mm, 3 μm, CERI). Mobile phases A, B, and C were 1 mmol/l of ammonium formate, acetonitrile, and 1 mmol/l of 2-propanol containing ammonium formate, respectively. The column oven temperature and flow rate were set to 40 °C and 0.3 ml/min, respectively. The sample was cooled to 4 °C and the injection volume was 3 μl. Mass scan range was 200-1,600 m/z, and spectra were acquired automatically in the data-dependent top N3 scan mode (positive/negative) with a resolution of 30,000. The QC sample was measured three times, and all sample solutions were measured in succession. Each QC sample was then measured three times. In addition, the QC sample was analyzed once per nine sample solutions.

The coefficient of variation of the peak area of the internal standard [PC (16: 0 D31-18: 1)] in the analysis of all QC samples was 5.3%, which was considered good reproducibility.

Lipid Search Ver.4.2 (Mitsui Knowledge Industry, Tokyo, Japan) was used for lipid molecular species identification and alignment process between samples (8). Peak picking, lipid molecular species estimation processing, and alignment processing were performed under the conditions shown in Supplementary Tables S2 and S3. Among the peaks estimated to be derived from lipids, those satisfying all conditions shown in Supplementary Table S3 were adopted. Supplementary Figure S4 shows a graphical representation of peak area values (raw values) for representative compounds, including the blank sample.

The peak area of the detected peak in each sample was normalized by the peak area of PC (16:0D31-18:1) as an internal standard and the creatinine concentration of each sample.

# DATA processing

Variables (metabolites) with >50% missing values were removed, and missing values were replaced by 1/5 of the minimum positive value for each compound using MetaboAnalysist, a web-based platform for metabolomics data analysis (MetaboAnalysist4.0, RRID:SCR_015539).

# References

1. Watanabe Y, Hirao Y, Kasuga K, Tokutake T, Semizu Y, Kitamura K, et al. Molecular Network Analysis of the Urinary Proteome of Alzheimer’s Disease Patients. Dementia Geriatric Cognitive Disord Extra (2019) 9:53–65. doi:10.1159/000496100

2. Soga T, Heiger DN. Amino Acid Analysis by Capillary Electrophoresis Electrospray Ionization Mass Spectrometry. Anal Chem (2000) 72:1236–1241. doi:10.1021/ac990976y

3. Soga T, Ueno Y, Naraoka H, Ohashi Y, Tomita M, Nishioka T. Simultaneous Determination of Anionic Intermediates forBacillussubtilisMetabolic Pathways by Capillary Electrophoresis Electrospray Ionization Mass Spectrometry. Anal Chem (2002) 74:2233–2239. doi:10.1021/ac020064n

4. Soga T, Ohashi Y, Ueno Y, Naraoka H, Tomita M, Nishioka T. Quantitative Metabolome Analysis Using Capillary Electrophoresis Mass Spectrometry. J Proteome Res (2003) 2:488–494. doi:10.1021/pr034020m

5. Sugimoto M, Wong DT, Hirayama A, Soga T, Tomita M. Capillary electrophoresis mass spectrometry-based saliva metabolomics identified oral, breast and pancreatic cancer-specific profiles. Metabolomics (2010) 6:78–95. doi:10.1007/s11306-009-0178-y

6. Ozaki H, Nakano Y, Sakamaki H, Yamanaka H, Nakai M. Basic eluent for rapid and comprehensive analysis of fatty acid isomers using reversed-phase high performance liquid chromatography/Fourier transform mass spectrometry. J Chromatogr A (2019) 1585:113–120. doi:10.1016/j.chroma.2018.11.057

7. Mori N, Fukano Y, Arita R, Shirakawa R, Kawazu K, Nakamura M, et al. Rapid identification of fatty acids and (O-acyl)-ω-hydroxy fatty acids in human meibum by liquid chromatography/high-resolution mass spectrometry. J Chromatogr A (2014) 1347:129–136. doi:10.1016/j.chroma.2014.04.082

8. Taguchi R, Ishikawa M. Precise and global identification of phospholipid molecular species by an Orbitrap mass spectrometer and automated search engine Lipid Search. J Chromatogr A (2010) 1217:4229–4239. doi:10.1016/j.chroma.2010.04.034
